# Characterization of the transactivation and nuclear localization functions of *Pichia pastoris* zinc finger transcription factor Mxr1p

**DOI:** 10.1016/j.jbc.2021.101247

**Published:** 2021-09-25

**Authors:** Aditi Gupta, Kamisetty Krishna Rao, Umakant Sahu, Pundi N. Rangarajan

**Affiliations:** Department of Biochemistry, Indian Institute of Science, Bangalore, India

**Keywords:** yeast metabolism, *Pichia pastoris*, Mxr1p, transcriptional regulation, aldehyde dehydrogenase, alcohol oxidase, transactivation domain, nuclear localization, 9aaTAD, nine-amino acid TAD, ACS, acetyl-CoA synthetase, ALD, aldehyde dehydrogenase, ALD6-1^Myc^, Myc-tagged ALD6-1, cDNA, complementary DNA, DBD, DNA-binding domain, GEO, Gene Expression Omnibus, GO, Gene Ontology, MUT, methanol utilization, MXRE, Mxr1p response element, NCBI, National Center for Biotechnology Information, NLS, nuclear localization signal, PGK, phosphoglycerate kinase, qPCR, quantitative PCR, TAD, transactivation domain, YNB, yeast nitrogen base, YNBE, YNB and 1% ethanol, YNBM, YNB and 1% methanol, YPD, yeast extract, peptone, and dextrose

## Abstract

The zinc finger transcription factor Mxr1p regulates the transcription of genes involved in methanol, acetate, and amino acid metabolism of the industrial yeast *Pichia pastoris* (*a.k.a. Komagataella phaffii*) by binding to Mxr1p response elements in their promoters. Here, we demonstrate that Mxr1p is a key regulator of ethanol metabolism as well. Using transcriptomic analysis, we identified target genes of Mxr1p that mediate ethanol metabolism, including *ALD6-1* encoding an aldehyde dehydrogenase. ALD6-1 is essential for ethanol metabolism, and the *ALD6-1* promoter harbors three Mxr1p response elements to which Mxr1p binds *in vitro* and activates transcription *in vivo*. We show that a nine-amino acid transactivation domain located between amino acids 365 and 373 of Mxr1p is essential for the transactivation of *ALD6-1* to facilitate ethanol metabolism. Mxr1N250, containing the N-terminal 250 amino acids of Mxr1p, localized to the nucleus of cells metabolizing ethanol dependent on basic amino acid residues present between amino acids 75 and 85. While the N-terminal 400 amino acids of Mxr1p are sufficient for the activation of target genes essential for ethanol metabolism, the region between amino acids 401 and 1155 was also required for the regulation of genes essential for methanol metabolism. Finally, we identified several novel genes whose expression is differentially regulated by Mxr1p during methanol metabolism by DNA microarray. This study demonstrates that Mxr1p is a key regulator of ethanol metabolism and provides new insights into the mechanism by which Mxr1p functions as a global regulator of multiple metabolic pathways of *P. pastoris*.

*Pichia pastoris* (*a.k.a. Komagataella phaffii*) is a methylotrophic yeast capable of utilizing methanol as a sole carbon source. The methanol utilization pathway enzyme alcohol oxidase, encoded by *AOX1*, constitutes 30% of the total soluble protein. This strong induction under methanol conditions has led to the development of a methanol-inducible *AOX1*-based promoter for commercial production of heterologous proteins (1, 2). It is a respiratory yeast, suggesting that in high glucose conditions biomass production through tricarboxylic acid cycle is favored over the conventional ethanol generation route followed in other yeasts like *Saccharomyces cerevisiae* because of the increased glycolytic flux. Consequently, the high biomass generation in the bioreactors dramatically increases the product yields, making them industrially more relevant (3). Another remarkable feature of this yeast is the ability to utilize a plethora of carbon compounds as the sole source of carbon. These include glucose, glycerol, ethanol, acetic acid, oleic acid, sorbitol, and amino acids such as glutamate. The genes encoding key enzymes of these metabolic pathways are primarily regulated at the transcriptional level (4, 5). In the case of methanol metabolism, several transcriptional regulators, such as Mxr1p, Trm1p, Mit1p, Rop1p, Mig1p, Mig2p, and Nrg1p, regulate the transcription of *AOX1* gene encoding alcohol oxidase 1 (6, 7, 8, 9, 10, 11). Mxr1p regulates the expression of genes of acetate and amino acid metabolism as well and thus functions as a global regulator of central carbon metabolism (4, 5). While Mxr1p primarily functions as a transcriptional activator, it is known to repress the expression of *GT1* encoding glycerol transporter during glycerol metabolism (12).

Transcriptional activators bind to DNA through their DNA-binding domain (DBD) and activate transcription by interacting with proteins of preinitiation complex, either directly or *via* coactivators through their transactivation domains (TADs) (13, 14). DBDs consist of motifs, such as zinc fingers, helix–turn–helix motif, or basic helix–loop–helix leucine zipper, whereas the TADs consist of amphipathic alpha helices, glutamine-rich domains, or proline-rich domains (15). Nuclear localization of transcription factors is facilitated by nuclear localization signals (NLSs), and the classical NLS is characterized by the presence of monopartite or bipartite NLSs consisting of one or two clusters of basic amino acids separated by a spacer, respectively (16). *P. pastoris* Mxr1p possesses a DBD in the amino terminus consisting of two C_2_H_2_ zinc fingers, which exhibit strong homology to the DBD of *S. cerevisiae* Adr1p (6, 17, 18). Mxr1p DBD binds to Mxr1p response elements (MXREs) in the promoters of target genes bearing the consensus sequence 5′-CYCCNY-3′ (4, 5, 18, 19). A TAD enriched in phenylalanine residues present between amino acid residues 246 and 280 of Mxr1p was shown to be functional during methanol metabolism but not ethanol metabolism (20). Serine 215 residue located upstream of this TAD is phosphorylated in cells metabolizing ethanol but not methanol, and interaction of this phosphoserine with 14-3-3 protein inhibits the function of the N-terminal TAD (20). Mxr1p is cytosolic in cells metabolizing glucose and localizes to the nucleus when cultured in media containing nonfermentable carbon sources (4, 6). Thus far, the NLS of Mxr1p has not been characterized.

In *S. cerevisiae*, ethanol is metabolized into acetaldehyde, acetate, and acetyl-CoA by the sequential action of alcohol dehydrogenase, aldehyde dehydrogenase (ALD), and acetyl-CoA synthetase (ACS), respectively. In *S. cerevisiae* as well as *Kluyveromyces lactis*, expression of alcohol dehydrogenase 2 is regulated by Adr1p, which is regarded as the homolog of Mxr1p (17, 21). However, the ability of *P. pastoris* Mxr1p to regulate genes of ethanol metabolism is not known. Considering the existing gaps, the present study was initiated to investigate the regulatory role of Mxr1p during ethanol metabolism, identify the NLS, and characterize the transactivation functions. Using high-throughput genome-wide RNA-Seq, we have identified novel target genes of Mxr1p essential for ethanol metabolism. Among these, *ALD6-1* encoding an ALD, which is downregulated in *Δmxr1*, was further characterized. We have identified an NLS and a nine-amino acid TAD (9aaTAD) in the amino terminal region of Mxr1p required for the regulation of genes of ethanol metabolism. Finally, we have identified several novel target genes of Mxr1p required for methanol metabolism using DNA microarray.

## Results

### Transcriptional regulation of ethanol metabolism by Mxr1p

**Figure 1:**
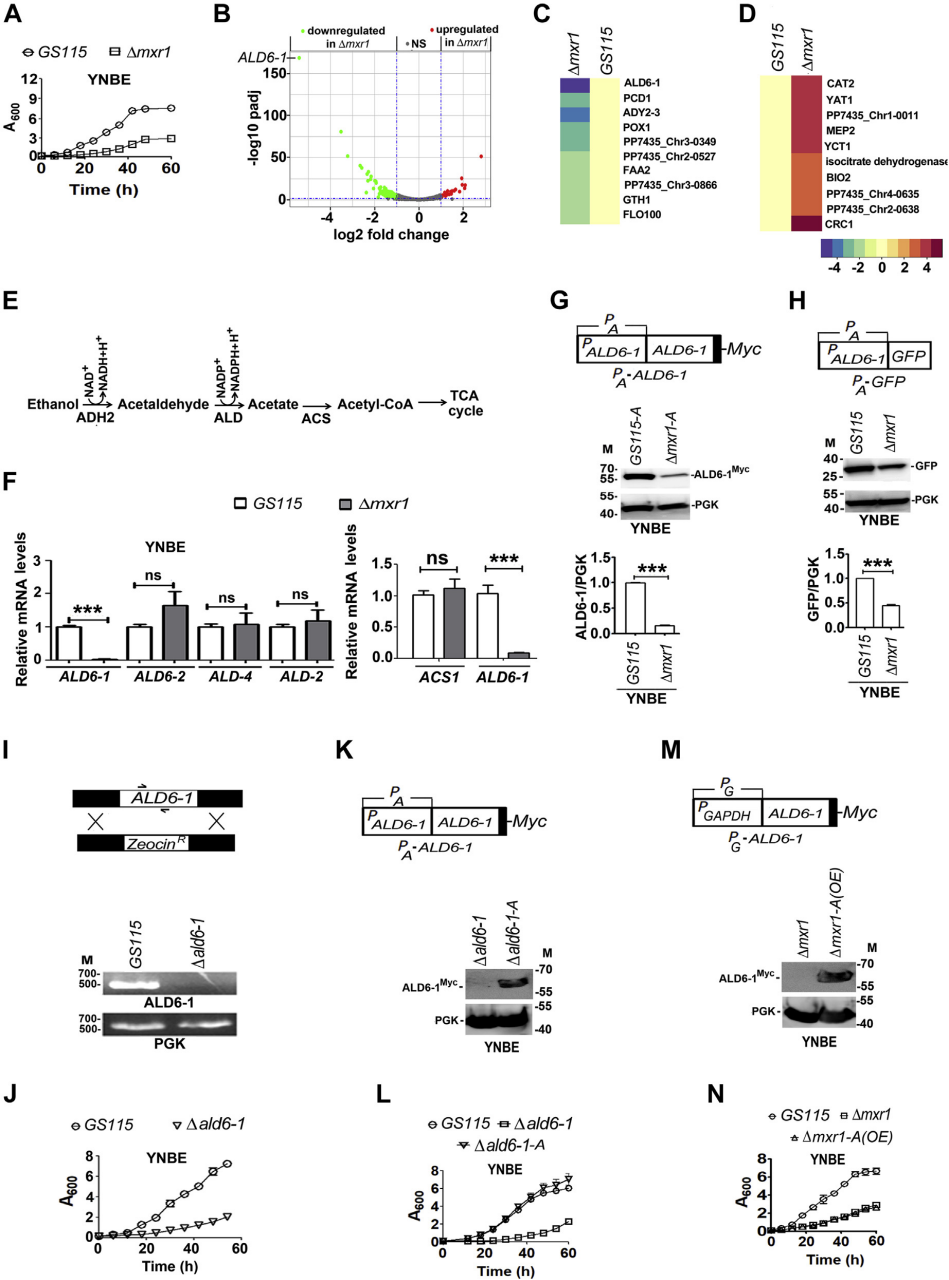
**Identification of target genes of Mxr1p during ethanol metabolism by RNA-Seq and characterization of *ALD6-1*.***A*, analysis of growth of *GS115* and *Δmxr1* cultured in YNBE. *B*, volcano plot of genes differentially regulated in *GS115* and *Δmxr1* cultured in YNBE. *ALD6-1* is the most downregulated gene in *Δmxr1*. *C*, heat map depicting genes that are highly downregulated in *Δmxr1*. *D*, heat map depicting genes that are highly upregulated in *Δmxr1*. *E*, schematic representation of ethanol utilization pathway of *Saccharomyces cerevisiae*. *F*, quantitation of *ALD* and *ACS1* mRNAs in *GS115* and *Δmxr1* cultured in YNBE by quantitative PCR (qPCR). *G*, analysis of expression of ALD6-1^Myc^ from 1.0-kb *ALD6-1* promoter (*P*_*A*_) in *GS115* and *Δmxr1* cultured in YNBE by Western blotting using anti-Myc antibodies. *H*, analysis of GFP expression from *P*_*A*_ in *GS115* and *Δmxr1* cultured in YNBE by Western blotting using anti-GFP antibodies. Quantitation of the data is presented. The intensity of individual bands was quantified and expressed as arbitrary units ± SD relative to controls (n = 3). *I*, strategy for the generation of *Δald6-1* and confirmation of deletion of *ALD6-1* by PCR. Gene-specific primers (*arrows*) amplify *ALD6-1* from genomic DNA of *GS115* but not *Δald6-1*. PGK served as control. *J*, analysis of growth of *GS115* and *Δald6-1* in YNBE. *K*, schematic representation of *P*_*A*_*-ALD6-1* construct and analysis of ALD6-1^Myc^ expression by Western blotting using anti-Myc antibodies. *L*, analysis of growth of different *Pichia pastoris* strains cultured in YNBE. *M*, schematic representation of *P*_*G*_*-ALD6-1* construct and analysis of ALD6-1^Myc^ expression by Western blotting using anti-Myc antibodies in cells cultured in YNBE. *N*, analysis of growth of different *P. pastoris* strains cultured in YNBE. Error bars in each figure indicate SD; n = 3. In the bar diagrams, *p* value summary is mentioned on the bar of each figure. ∗*p* < 0.05, ∗∗*p* < 0.005, ∗∗∗*p* < 0.0005, and ns. Student's paired or unpaired *t* test was done. In Western blots, M indicates molecular weight markers (kilodalton). ACS, acetyl-CoA synthetase; ALD, aldehyde dehydrogenase; ns, not significant; PGK, phosphoglycerate kinase; YNBE, YNB and 1% ethanol.

*P. pastoris* strains used in this study are listed in Table 1. To examine the role of Mxr1p in the regulation of genes of ethanol metabolism, *GS115* and *Δmxr1* were cultured in a medium containing yeast nitrogen base (YNB) and 1% ethanol (YNBE), and their growth pattern was studied. Growth of *Δmxr1* was significantly retarded in comparison with that of *GS115* (Fig. 1*A*) suggesting that Mxr1p regulates the expression of key genes of ethanol metabolism required for normal growth of *P. pastoris*. To gain insights into the mechanism of transcriptional regulation of genes of ethanol metabolism by Mxr1p, genome-wide RNA-Seq was carried out. *GS115* and *Δmxr1* were cultured in YNBE, RNA was isolated, and biological replicates were subjected to RNA-Seq. The datasets have been deposited in the Gene Expression Omnibus (GEO) database under accession identification number GSE168677. A total of 25 to 30 million reads were obtained for each sample after quality trimming. Samples were aligned to the *K. phaffii* strain CBS 7435 reference genome (https://www.ncbi.nlm.nih.gov/assembly/GCA_900235035.1/). Differentially expressed genes were identified by normalizing the read counts in *Δmxr1* to that of *GS115* (control). Deletion of *MXR1* resulted in significant changes in the transcriptome. Of 5424 genes, 170 were downregulated and 28 were upregulated in *Δmxr1* (Supporting information 2). For this analysis, the threshold for statistical significance was considered an adjusted *p* value of <0.05 and log2 fold change of ±1 for upregulated and downregulated genes. The overall change and the most highly upregulated and downregulated genes are shown in the volcano plot and heat maps, respectively (Fig. 1, *B*–*D*). *ALD6-1*, encoding an ALD, was the most downregulated gene in *Δmxr1* (greater than fivefold). Other genes downregulated in *Δmxr1* include those encoding peroxisomal proteins, PP7435_Chr3-0349 encoding a transcription factor homologous to *S. cerevisiae YLL054C* (https://www.yeastgenome.org/locus/S000003977), PP7435_Chr2-0527 encoding glutathione transferase involved in detoxification of hydrogen peroxide, lipid metabolism genes (*POX1*, *FAA2*, and *PCD1* encoding acyl Co-A oxidase, long-chain fatty acyl CoA synthetase, and pyrophosphatase, respectively) and *GTH1* encoding a high-affinity glucose transporter. Interestingly, *ADY2-3* encoding exporter of ammonia was downregulated, whereas MEP2 encoding ammonia transporter was upregulated in *Δmxr1*. *CRC1*, *CAT2*, and *YAT1* encoding proteins involved in carnitine transport were highly upregulated in *Δmxr1*. Genes encoding acetyl CoA metabolizing enzymes, such as isocitrate lyase, isocitrate dehydrogenase, and isocitrate/isopropylmalate dehydrogenase, were also upregulated in *Δmxr1* probably to facilitate metabolism of acetyl CoA *via* tricarboxylic acid cycle to generate energy in the absence of *ALD6-1*, the ethanol-metabolizing gene.

**Table 1:** *Pichia pastoris* strains used in this study

Strain	Description	References
*GS115*	*his4*	(6)
*Δmxr1*	*GS115, Ppmxr1Δ::Zeo*^*r*^	(6)
*GS115-A*	*GS115, HIS4::(P*_*A*_*PpALD6-1-Myc)*	This study
*Δmxr1-A*	*Δmxr1, HIS4::(P*_*A*_*PpALD6-1-Myc)*	This study
*GS115-P*_*A*_*-GFP*	*GS115, HIS4::(P*_*A*_*-GFP)*	This study
*Δmxr1-P*_*A*_*-GFP*	*Δmxr1, HIS4::(P*_*A*_*-GFP)*	This study
*Δald6-1*	*GS115, Ppald6-1Δ::Zeo*^*r*^	This study
*Δald6-1-A*	*Δald6-1, HIS4::(P*_*A*_*PpALD6-1-Myc)*	This study
*Δmxr1-A(OE)*	*Δmxr1, HIS4::(P*_*GAPDH*_*PpALD6-1-Myc)*	This study
*GS115-P*_*A-M1*_*-GFP*	*GS115, HIS4::(P*_*A-M1*_*-GFP)*	This study
*GS115-P*_*A-M2*_*-GFP*	*GS115, HIS4::(P*_*A-M2*_*-GFP)*	This study
*GS115-P*_*A-M3*_*-GFP*	*GS115, HIS4::(P*_*A-M3*_*-GFP)*	This study
*GS115-P*_*A-M4*_*-GFP*	*GS115, HIS4::(P*_*A-M4*_*-GFP)*	This study
*Δmxr1-FL*	*Δmxr1, Bla*^*r*^*(P*_*GAPDH*_*PpMXR1-Myc)*	(4)
*Δmxr1-N400*	*Δmxr1, Bla*^*r*^*(P*_*GAPDH*_*PpMXR1N400-Myc)*	(4)
*Δmxr1-A-N400*	*Δmxr1-A, Bla*^*r*^*(P*_*GAPDH*_*PpMXR1N400-Myc)*	This study
*Δmxr1-A-N400F∗*	*Δmxr1-A, Hyg*^*r*^*(P*_*GAPDH*_*-PpMXR1N400F∗-Myc)*	This study
*Δmxr1-A-N400Q∗*	*Δmxr1-A, Hyg*^*r*^*(P*_*GAPDH*_*-PpMXR1N400Q∗-Myc)*	This study
*Δmxr1-A-N400F∗Q∗*	*Δmxr1-A, Hyg*^*r*^*(P*_*GAPDH*_*-PpMXR1N400F∗Q∗-Myc)*	This study
*Δmxr1-A-N250*	*Δmxr1-A, Hyg*^*r*^*(P*_*GAPDH*_*GFP-PpMXR1N250)*	This study
*Δmxr1-A-N150*	*Δmxr1-A, Hyg*^*r*^*(P*_*GAPDH*_*GFP-PpMXR1N150)*	This study
*Δmxr1-N250*	*Δmxr1, Hyg*^*r*^*(P*_*GAPDH*_*GFP-PpMXR1N250)*	This study
*Δmxr1-N400ΔTAD*	*Δmxr1, Hyg*^*r*^*(P*_*GAPDH*_*PpMXR1N400ΔTAD-Myc)*	This study
*Δmxr1-N62*	*Δmxr1, Hyg*^*r*^*(P*_*GAPDH*_*GFP-PpMXR1N62)*	This study
*Δmxr1-N81*	*Δmxr1, Hyg*^*r*^*(P*_*GAPDH*_*GFP-PpMXR1N81)*	This study
*Δmxr1-N109*	*Δmxr1, Hyg*^*r*^*(P*_*GAPDH*_*GFP-PpMXR1N109)*	This study
*Δmxr1-N250-M2*	*Δmxr1, Hyg*^*r*^*(P*_*GAPDH*_*GFP-PpMXR1N250-M2)*	This study
*Δmxr1-N250-M1*	*Δmxr1, Hyg*^*r*^*(P*_*GAPDH*_*GFP-PpMXR1N250-M1)*	This study

### *ALD6-1* is indispensable for ethanol metabolism

In *S. cerevisiae*, ALDs catalyze the conversion of acetaldehyde to acetate, the second step of ethanol metabolism (Fig. 1*E*). Since *ALD6-1* is the most downregulated gene in *Δmxr1* cultured in YNBE, we investigated its function and regulation in detail. *P. pastoris* genome harbors four *ALD* genes annotated as *ALD6-1*, *ALD6-2*, *ALD4*, and *ALD2* (Table 2). Quantitation of the four *ALD* mRNAs by quantitative PCR (qPCR) indicated that *ALD6-1* alone was downregulated in *Δmxr1* during ethanol metabolism (Fig. 1*F*). Since *ACS1* encoding ACS1 was shown to be downregulated in *Δmxr1* during acetate metabolism (4), we examined *ACS1* transcript levels in cells metabolizing ethanol. The results indicate that *ACS1* mRNA levels are comparable in *GS115* and *Δmxr1* during ethanol metabolism (Fig. 1*F*) suggesting that Mxr1p does not regulate *ACS1* expression during ethanol metabolism. It is pertinent to mention that *P. pastoris* homologs of transcription factor Cat8 regulate the expression of *ACS1* during ethanol metabolism (22). To further investigate regulation of *ALD6-1* by Mxr1p, we generated *GS115-A* and *Δmxr1-A* expressing Myc-tagged ALD6-1 (ALD6-1^Myc^) and examined ALD6-1 levels in the lysates of cells cultured in YNBE by Western blotting using anti-Myc epitope antibodies. The results indicate that ALD6-1^Myc^ is downregulated in *Δmxr1-A* (Fig. 1*G*). The gene encoding GFP was cloned downstream of 1.0 kb of *ALD6-1* promoter (*P*_*A*_*-GFP*), and protein levels in the lysates of *GS115* and *Δmxr1* cultured in YNBE were examined by Western blotting using anti-GFP antibodies. GFP levels are also downregulated in *Δmxr1* (Fig. 1*H*) suggesting that Mxr1p is likely to regulate *ALD6-1* expression at the transcriptional level. To understand the importance of *ALD6-1* in ethanol metabolism, gene encoding ALD6-1 was deleted to generate *Δald6-1* (Fig. 1*I*), which exhibits retarded growth when cultured in YNBE (Fig. 1*J*). Expression of ALD6-1^Myc^ from its own promoter in *Δald6-1* (Fig. 1*K*) readily restores growth in YNBE (Fig. 1*L*). However, expression of ALD6-1^Myc^ from *GAPDH* promoter in *Δmxr1* (Fig. 1*M*) does not result in the restoration of growth in YNBE (Fig. 1*N*). Thus, *ALD6-1* has a unique, essential, and nonredundant function during ethanol metabolism. The ability of ALD6-1 to restore growth of *Δald6-1* but not *Δmxr1* suggests that Mxr1p-regulated genes other than *ALD6-1* (Fig. 1, *C* and *D*) are essential for ethanol metabolism.

**Table 2:** *Pichia pastoris ALD*s

Gene	*P. pastoris CBS7435*	UniProt ID	*P. pastoris GS115*	UniProt ID
*ALD6-1*	PP7435_Chr4-0972	F2R0E4	PAS_chr4_0043	C4R6P6
*ALD6-2*	PP7435_Chr3-0183	F2QUS7	PAS_chr3_0987	C4R664
*ALD4*	PP7435_Chr2-0787	F2QSU4	PAS_chr2-1_0853	C4R0W4
*ALD2*	PP7435_Chr2-0843	F2QSZ7	PAS_chr2-1_0453	C4R0Q6

### Identification of MXREs in *ALD6-1* promoter (*P*_*A*_)

**Figure 2:**
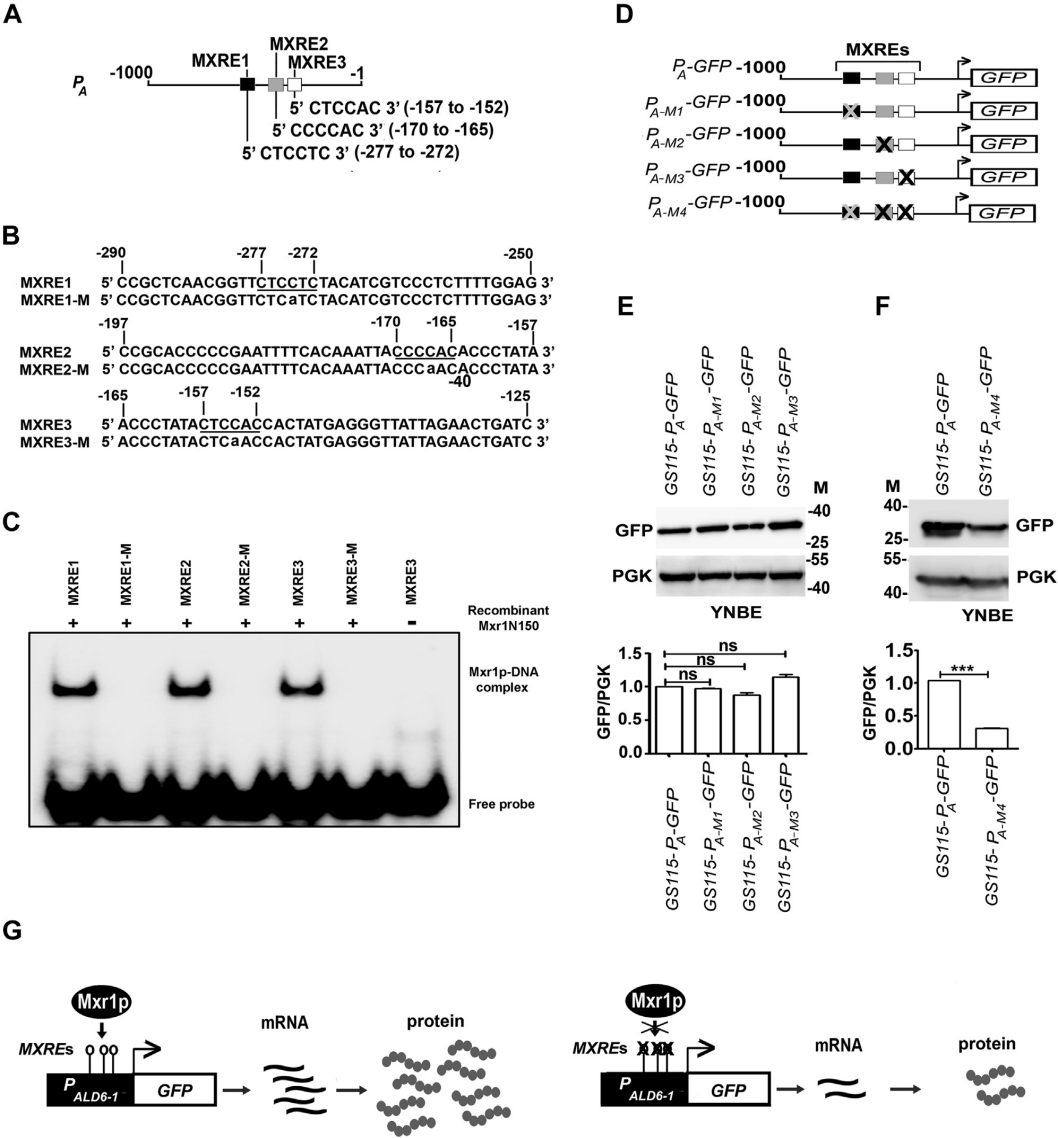
**Transcriptional regulation of *ALD6-1* by Mxr1p and importance of MXREs in *ALD6-1* promoter (*P***_***A***_**).***A*, schematic representation of position of putative MXREs in *ALD6-1* promoter. *B*, nucleotide sequence of oligonucleotides used in EMSA. MXREs are *underlined*. In MXRE1-M, MXRE2-M, and MXRE3-M, 5′-CYCC-3′ was mutated to 5′-CYCA-3′. Mutated base is shown in *lower case*. *C*, analysis of recombinant Mxr1pN150 binding to radiolabeled *ALD6-1* promoter regions by EMSA. *D*, schematic representation of different *P*_*A*_*-GFP* constructs carrying point mutations in only one (*P*_*A-M1*_*-GFP*, *P*_*A-M2*_*-GFP*, and *P*_*A-M3*_*-GFP*) or all the three (*P*_*A-M4*_*-GFP*) MXREs. MXREs are boxed, and mutant MXREs are denoted by “X.” *E* and *F*, analysis of GFP expression from *P*_*A*_ containing wildtype or mutant MXREs by Western blotting using anti-GFP antibodies in lysates of cells cultured in YNBE. PGK was used as a loading control. M, protein molecular weight markers (kilodalton). The *panel* below shows quantitation of the Western blot data. The intensity of individual bands was quantified and expressed as arbitrary units ± SD relative to controls (n = 3). *F*, the faster migrating GFP band was also included during quantitation. *G*, schematic representation of regulation of *ALD6-1* by Mxr1p during ethanol metabolism. MXRE, Mxr1p response element; PGK, phosphoglycerate kinase.

Since Mxr1p regulates the transcription of target genes by binding to MXREs in their promoters bearing the consensus sequence 5′-CYCCNY-3′ (4, 5), we analyzed 1.0 kb of *ALD6-1* promoter (*P*_*A*_) and identified three putative MXREs designated as MXRE1, MXRE2, and MXRE3 (Fig. 2*A*). Since point mutations within the 5′-CYCCNY-3′ motif of *AOX1* MXREs abrogate Mxr1p binding (19), 5′-CYCC-3′ sequence was mutated to 5′-CYCA-3′ in the *P*_*A*_MXREs (Fig. 2*B*). Oligonucleotides carrying these mutations were synthesized and designated as MXRE1-M, MXRE2-M, and MXRE3-M (Fig. 2*B*). A recombinant Mxr1p containing 150 N-terminal amino acids including the DBD, which specifically binds to MXREs of *AOX1* promoter, was purified from *Escherichia coli* cell lysates (19) and used in an electrophoretic mobility shift assay together with radiolabeled *P*_*A*_MXREs. The results indicate that Mxr1pN150 binds to wildtype but not the mutant *P*_*A*_MXREs (Fig. 2*C*). *P*_*A-*_*GFP* constructs carrying a point mutation in one or all three MXREs were generated (Fig. 2*D*), transformed into *GS115*, and GFP levels were examined by Western blotting using anti-GFP antibodies in the lysates of cells cultured in YNBE. The results indicate that mutation of MXRE1, MXRE2, or MXRE3 alone has no significant effect on GFP expression (Fig. 2*E*). However, GFP expression is significantly reduced when all the three are mutated (Fig. 2*F*) suggesting that a combination of two MXREs is sufficient for transactivation by Mxr1p from *P*_*A*_ during ethanol metabolism. Key results obtained thus far are summarized in Figure 2*G*.

### Mxr1N400 activates *ALD6-1* transcription during ethanol metabolism

**Figure 3:**
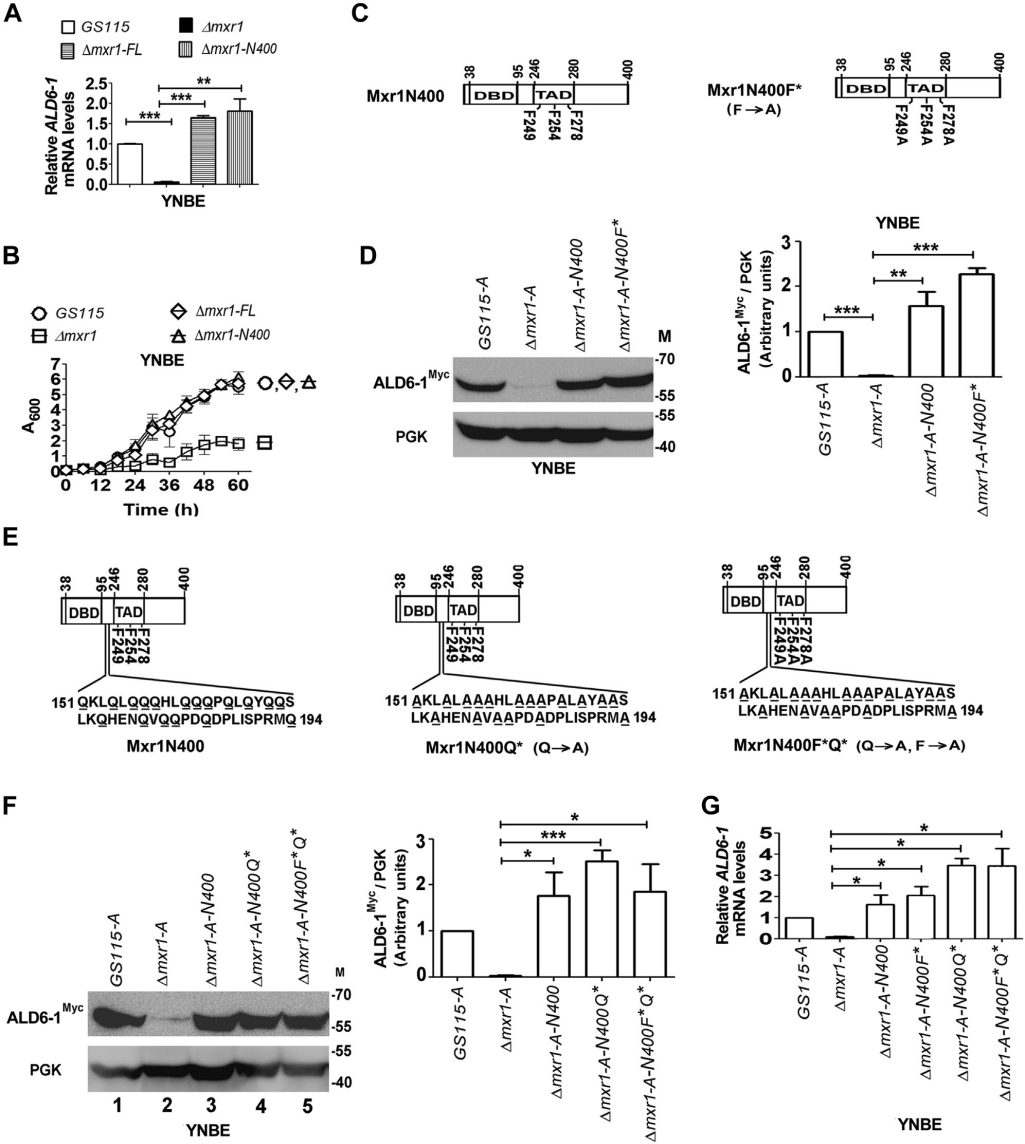
**Characterization of Mxr1N400 TAD during ethanol metabolism.***A*, quantitation of *ALD6-1* mRNA levels by quantitative PCR (qPCR) in different *Pichia pastoris* strains cultured in YNBE as indicated. *B*, analysis of growth of different *P. pastoris* strains cultured in YNBE. Absorbance at 600 nm of liquid cultures was measured at different time intervals as indicated. *C*, schematic representation of Mxr1N400 and Mxr1N400F∗. The TAD between amino acids 246 and 280 as well as F249, F254, F278, important for transactivation of *AOXI* as reported by Parua *et al.* (20), are shown. In Mxr1N400F∗, phenylalanine residues were mutated to alanine residues. *D*, Western blot analysis of ALD6-1^Myc^ using anti-Myc epitope antibodies in the lysates of different *P. pastoris* strains as indicated. Cells were cultured in YNBE for 14 h. Quantitation of data is shown. The intensity of individual bands was quantified and expressed as arbitrary units ± SD relative to controls (n = 3). *E*, schematic representation of Mxr1N400, Mxr1N400Q∗, and Mxr1N400F∗Q∗. Glutamine residues were mutated to alanine residues in Mxr1N400Q∗. Both glutamine and phenylalanine residues were mutated to alanine residues in Mxr1N400F∗Q∗. *F*, Western blot analysis of ALD6-1^Myc^ using anti-Myc epitope antibodies in the lysates of different *P. pastoris* strains as indicated. Cells were cultured in YNBE for 14 h. Quantitation of data is shown. The intensity of individual bands was quantified and expressed as arbitrary units ± SD relative to controls (n = 3). *G*, quantitation of *ALD6-1* mRNA levels by qPCR in different *P. pastoris* strains cultured in YNBE as indicated. Error bars in each figure indicate SD; n = 3. In the bar diagrams, *p* value summary is mentioned on the bar of each figure. ∗*p* < 0.05; ∗∗*p* < 0.005; ∗∗∗*p* < 0.0005; and ns. Student's unpaired *t* test was done. ns, not significant; TAD, transactivation domain; YNBE, yeast nitrogen base and 1% ethanol.

Among the various *P. pastoris* transcription factors identified thus far (6, 7, 8, 9, 10, 11), Mxr1p has the unique distinction of functioning as a global regulator of multiple metabolic pathways (4, 5, 12). Mxr1p full-length protein (Mxr1FL) consists of 1155 amino acids. A truncated Mxr1p (Mxr1N400) consisting of 400 N-terminal amino acids was shown to activate the transcription of Mxr1p target genes of acetate and amino acid metabolism as well as *AOX1* of methanol metabolism (4, 5, 20). Here, we examined the ability of Mxr1N400 to activate *ALD6-1* transcription during ethanol metabolism. When expressed in *Δmxr1*, Mxr1N400 was as efficient as Mxr1FL in restoring *ALD6-1* mRNA levels (Fig. 3*A*). Furthermore, Mxr1N400 reversed the growth defect of *Δmxr1* as efficiently as Mxr1FL (Fig. 3*B*). Thus, 400 N-terminal amino acids of Mxr1p are sufficient to activate the transcription of not only *ALD6-1* but also other Mxr1p-regulated genes necessary for ethanol metabolism.

Mxr1N400 was shown to possess a TAD between amino acid residues 246 and 280 (Fig. 3*C*) (20). Mutation of three phenylalanine residues (F249, F254, and F278) within this TAD to alanine has been reported to result in the loss of transactivation function of Mxr1p (20). To examine the role of these phenylalanine residues in the activation of *ALD6-1* transcription during ethanol metabolism, we generated Mxr1N400F∗ in which phenylalanine residues were mutated to alanine (Fig. 3*C*). When expressed in *Δmxr1-A*, it restores ALD6-1^Myc^ levels as efficiently as Mxr1N400 (Fig. 3*D*) indicating that phenylalanine residues are not essential for the transactivation of *ALD6-1* during ethanol metabolism.

Analysis of amino acid sequence of Mxr1N400 indicated the presence of a glutamine-rich region between 151 and 194 residues (Fig. 3*E*). Several transcription factors ranging from yeast to humans have been shown to contain glutamine-rich regions within their TADs (15, 23, 24, 25, 26, 27). To investigate the role of glutamine residues of Mxr1N400 in the transactivation of *ALD6-1*, we generated Mxr1N400Q∗ as well as Mxr1N400F∗Q∗ in which glutamine residues alone or glutamine as well as phenylalanine residues were mutated to alanine, respectively (Fig. 3*E*). When expressed in *Δmxr1-A*, both these mutant proteins restore ALD6-1^Myc^ protein (Fig. 3*F*) as well as mRNA (Fig. 3*G*) levels in cells cultured in YNBE. Thus, neither phenylalanine residues nor glutamine residues are essential for the transactivation of *ALD6-1* during ethanol metabolism.

**Figure 4:**
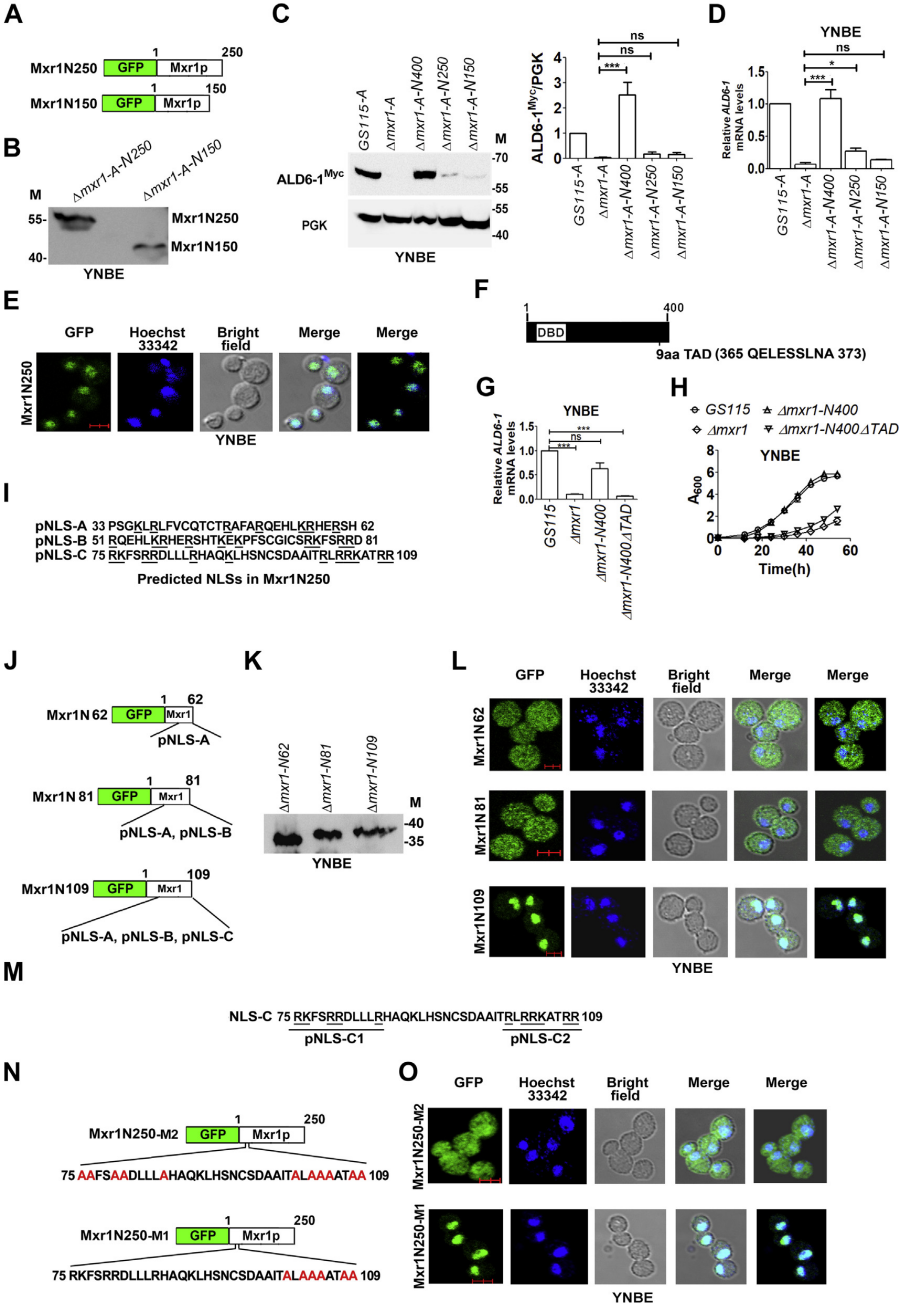
**Characterization of putative N-terminal TAD and NLS in Mxr1N250.***A*, schematic representation of Mxr1N250 and Mxr1N150 carrying N-terminal GFP fusions. *B*, Western blot analysis of Mxr1N250 and Mxr1N150 in cells cultured in YNBE using anti-GFP antibodies. *C*, Western blot analysis of ALD6-1^Myc^ in the lysates of different *Pichia pastoris* strains as indicated using anti-Myc epitope antibodies. Cells were cultured in YNBE for 14 h. Quantitation of data is shown. The intensity of individual bands was quantified and expressed as arbitrary units ± SD relative to controls (n = 3). *D*, quantitation of *ALD6-1* mRNA levels by quantitative PCR (qPCR) in different *P. pastoris* strains cultured in YNBE for 14 h. *E*, live cell confocal imaging of Mxr1N250 cultured in YNBE. Hoechst 33342 was used to stain nuclei. GFP was visualized by direct fluorescence. *F*, schematic representation of putative nine-amino acid TAD present in Mxr1N400 between amino acids 365 and 373. *G*, quantitation of *ALD6-1* mRNA levels by qPCR in different *P. pastoris* strains cultured in YNBE for 14 h. *H*, analysis of growth of different *P. pastoris* strains cultured in YNBE. *I*, three putative NLSs (pNLS-A, pNLS-B, and pNLS-C) in Mxr1N250 as predicted by cNLS mapper. *J*, schematic representation of Mxr1N62, Mxr1N81, and Mxr1N109 carrying N-terminal GFP fusions. *K*, Western blot analysis of Mxr1N62, Mxr1N81, and Mxr1N109 in cells cultured in YNBE using anti-GFP antibodies. *L*, live cell confocal imaging of Mxr1N62, Mxr1N81, and Mxr1N109 cultured in YNBE. Hoechst 33342 was used to stain nuclei. GFP was visualized by direct fluorescence. *M*, depiction of two clusters of basic amino acids (pNLS-C1 and pNLS-C2) in pNLS-C. *N*, mutation of basic amino acid residues in both pNLS-C1 and pNLS-C2 (Mxr1N250-M2) or pNLS-C2 alone (Mxr1N250M1) to alanine. *O*, subcellular localization of Mxr1N250-M2 and Mxr1N250-M1 was examined by direct GFP fluorescence as indicated. *C* and *D*, error bars indicate SD; n = 3. *p* Value summary is mentioned on the bar of each figure. ∗*p* < 0.05, ∗∗*p* < 0.005, ∗∗∗*p* < 0.0005, and ns. Student's unpaired *t* test was done. The scale bar in *E*, *L*, and *O* corresponds to 2.0 μm. NLS, nuclear localization signal; ns, not significant; TAD, transactivation domain; YNBE, yeast nitrogen base and 1% ethanol.

To further characterize the N-terminal TAD, we generated Mxr1N250 and Mxr1N150 constructs in which GFP was fused to 250 and 150 N-terminal amino acids of Mxr1p, respectively (Fig. 4*A*), and these were expressed in *Δmxr1-A*. Protein expression was confirmed by Western blotting using anti-GFP antibodies (Fig. 4*B*). Mxr1N250 and Mxr1N150 failed to restore ALD6-1^Myc^ protein (Fig. 4*C*) and mRNA (Fig. 4*D*) levels in *Δmxr1-A* suggesting that the region between 251 and 400 amino acid residues of Mxr1p harbors putative TAD(s) essential for ethanol metabolism. Nuclear localization is essential for the regulation of expression of target genes by transcription factors, and it is possible that the inability of Mxr1N250 to activate *ALD6-1* transcription may be due to the loss of an NLS present between 251 and 400 amino acids. To rule out this possibility, subcellular localization of Mxr1N250 was examined in cells cultured in YNBE by visualizing direct GFP fluorescence in a confocal microscope. The results indicate that Mxr1N250 localizes to the nucleus during ethanol metabolism (Fig. 4*E* and Fig. S1), and the inability of Mxr1N250 to activate *ALD6-1* expression may be due to the loss of a transactivation function present between 251 and 400 amino acids.

Transcription factors, such as Oaf1, Pip2, and Gal4, possess a 9aaTAD, which is present in several other transcription factors, such as E2A, MLL, p53, NF-κB, NFAT, CEBP, Sox18, Pdr1, Gcn4, and VP16 (28, 29, 30, 31). The 9aaTAD interacts with the transcriptional cofactor TAF9 as well as the KIX domain of general transcriptional mediators CBP, p300, or MED15 (28, 29, 30, 31). *In silico* analysis of the amino acid sequence of Mxr1N400 using the 9aaTAD prediction tool (https://www.med.muni.cz/9aaTAD/index.php) indicated the presence of a putative 9aaTAD between amino acids 365 and 373 (Fig. 4*F*). To examine its function, Mxr1N400 carrying a deletion of this putative TAD was generated (Mxr1N400ΔTAD), expressed in *Δmxr1*, and *ALD6-1* mRNA levels were analyzed by qPCR. The results indicate that Mxr1N400 but not Mxr1N400ΔTAD restores *ALD6-1* mRNA levels (Fig. 4*G*) as well as growth (Fig. 4*H*) of *Δmxr1* indicating that the putative 9aaTAD functions as TAD during ethanol metabolism.

### Identification of NLS in Mxr1N250

The localization of Mxr1N250 in the nucleus of cells cultured in YNBE led us to investigate the presence of NLS within 250 N-terminal amino acids. Analysis of the amino acid sequence of Mxr1N250 by cNLS mapper (http://nls-mapper.iab.keio.ac.jp/cgi-bin/NLS_Mapper_form.cgi) revealed the presence of multiple putative NLSs (pNLS-A, pNLS-B, and pNLS-C) (Fig. 4*I*). To examine their function, 62 (Mxr1N62), 81 (Mxr1N81), and 109 (Mxr1N109) N-terminal amino acids of Mxr1p were fused to GFP (Fig. 4*J*) and expressed in *Δmxr1* (Fig. 4*K*). Subcellular localization studies indicate that Mxr1N62 and Mxr1N81 are cytosolic, whereas Mxr1N109 localizes to the nucleus (Fig. 4*L* and Figs. S2–S4) indicating that pNLS-C harbors an NLS. pNLS-C resembles a classical bipartite NLS (16) consisting of two clusters of basic amino acids (pNLS-C1 and pNLS-C2) separated by a spacer (Fig. 4*M*). Basic amino acid residues in pNLS-C1 as well as pNLS-C2 (Mxr1N250-M2) or pNLS-C2 alone (Mxr1N250-M1) were mutated to alanine residues (Fig. 4*N*), and nuclear localization was examined in cells cultured in YNBE. Mxr1N250-M1 but not Mxr1N250-M2 localizes to the nucleus (Fig. 4*O*, Figs. S5, and S6) indicating that the pNLS-C1 functions as monopartite NLS in Mxr1N250.

### Mxr1N400 is necessary but not sufficient for the activation of Mxr1p target genes required for methanol metabolism

**Figure 5:**
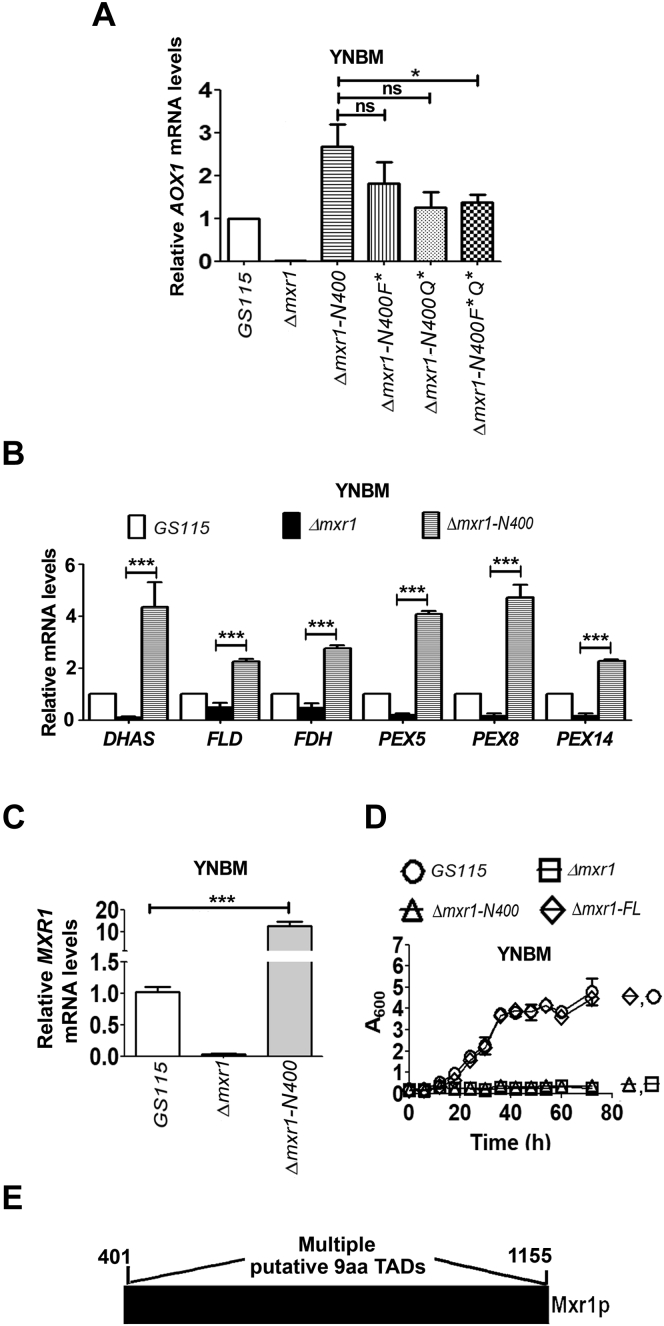
**Analysis of the role of Mxr1N400 and Mxr1FL in methanol metabolism.***A*, quantitation of *AOXI* mRNA by quantitative PCR (qPCR) during methanol metabolism in different *Pichia pastoris* strains as indicated. Cells were cultured in YNBM for 14 h. Error bars in each figure indicate SD; n = 3. *B* and *C*, quantitation of *DHAS*, *FLD*, *FDH*, *PEX5*, *PEX8*, *PEX14*, as well as *MXR1* mRNAs by qPCR during methanol metabolism in different *P. pastoris* strains as indicated. Error bars in each figure indicate SD; n = 3. *D*, analysis of growth of different *P. pastoris* strains cultured in YNBM. *E*, schematic diagram depicting putative nine-amino acid TADs between amino acids 401 and 1155 of Mxr1p. For details, see Fig. S7. In the bar diagrams, *p* value summary is mentioned on the bar of each figure. ∗*p* < 0.05, ∗∗*p* < 0.005, ∗∗∗*p* < 0.0005, and ns. Student's paired or unpaired *t* test was done. ns, not significant; YNBM, yeast nitrogen base and 1% methanol.

During methanol metabolism, Mxr1p activates the transcription of *AOX1* encoding alcohol oxidase 1 as well as other methanol utilization (MUT) pathway genes, such as *DHAS*, *FLD*, *FDH*, *PEX5*, *PEX8*, and *PEX14* encoding dihydroxyacetone synthase, formaldehyde dehydrogenase, formate dehydrogenase, peroxins 5, 8, and 14, respectively (6). Furthermore, Mxr1N400 was shown to be sufficient for the activation of *AOX1* during methanol metabolism (20). Interestingly, Mxr1N400 as well as Mxr1N400F∗, MxrN400Q∗, and Mxr1N400F∗Q∗ restore *AOX1* mRNA levels when expressed in *Δmxr1* cultured in YNB and 1% methanol (YNBM) (Fig. 5*A*). Thus, phenylalanine and glutamine residues in Mxr1N400 have no major role in the activation of Mxr1p target genes of ethanol as well as methanol metabolism (Figs. 3*G* and 5*A*). However, the ability of Mxr1N400 to regulate transcription of Mxr1p target genes other than *AOX1* has not been investigated. Here, we demonstrate that Mxr1N400 activates the transcription of not only *AOX1* but also other MUT pathway genes as evident from qPCR analysis of RNA isolated from cells cultured in a medium containing YNBM (Fig. 5*B*). The mRNA levels of MUT pathway genes are twofold to fivefold higher in *Δmxr1-N400* than those in *GS115* because of overproduction of Mxr1N400 from *GAPDH* promoter as evident from qPCR analysis of *MXR1* mRNA levels (Fig. 5*C*). Surprisingly, Mxr1N400 does not rescue the growth defect of *Δmxr1* cultured in YNBM, despite activating the transcription of several key Mxr1p target genes of methanol metabolism (Fig. 5*D*). Thus, in addition to the known Mxr1p targets, that is, MUT pathway genes, Mxr1p regulates the expression of several other genes essential for methanol metabolism whose identity is not known. It is likely that transactivation of these essential genes is facilitated by other putative TAD(s) localized beyond 400 N-terminal amino acids of Mxr1p. Interestingly, the region between 401 and 1155 amino acids harbors several putative 9aaTADs (Fig. 5*E* and Fig. S7), some of which may be involved in the activation of genes essential for methanol metabolism.

### Identification of novel targets of Mxr1p essential for methanol metabolism

To identify genes of methanol metabolism whose transactivation is dependent on Mxr1FL rather than Mxr1N400, RNA was isolated from *GS115*, *Δmxr1*, *Δmxr1-FL*, and *Δmxr1-N400* grown in YNBM, and biological replicates were subjected to DNA microarray for the identification of differentially expressed genes. In all the comparisons, *GS115* was used as a control for clustering differentially expressed genes. To identify significantly regulated genes, a cutoff of *p* value <0.01 was applied. Microarray datasets have been deposited into the GEO database under accession identification number GSE146829. Volcano plots and heat maps are used to illustrate overall changes in gene expression.

**Figure 6:**
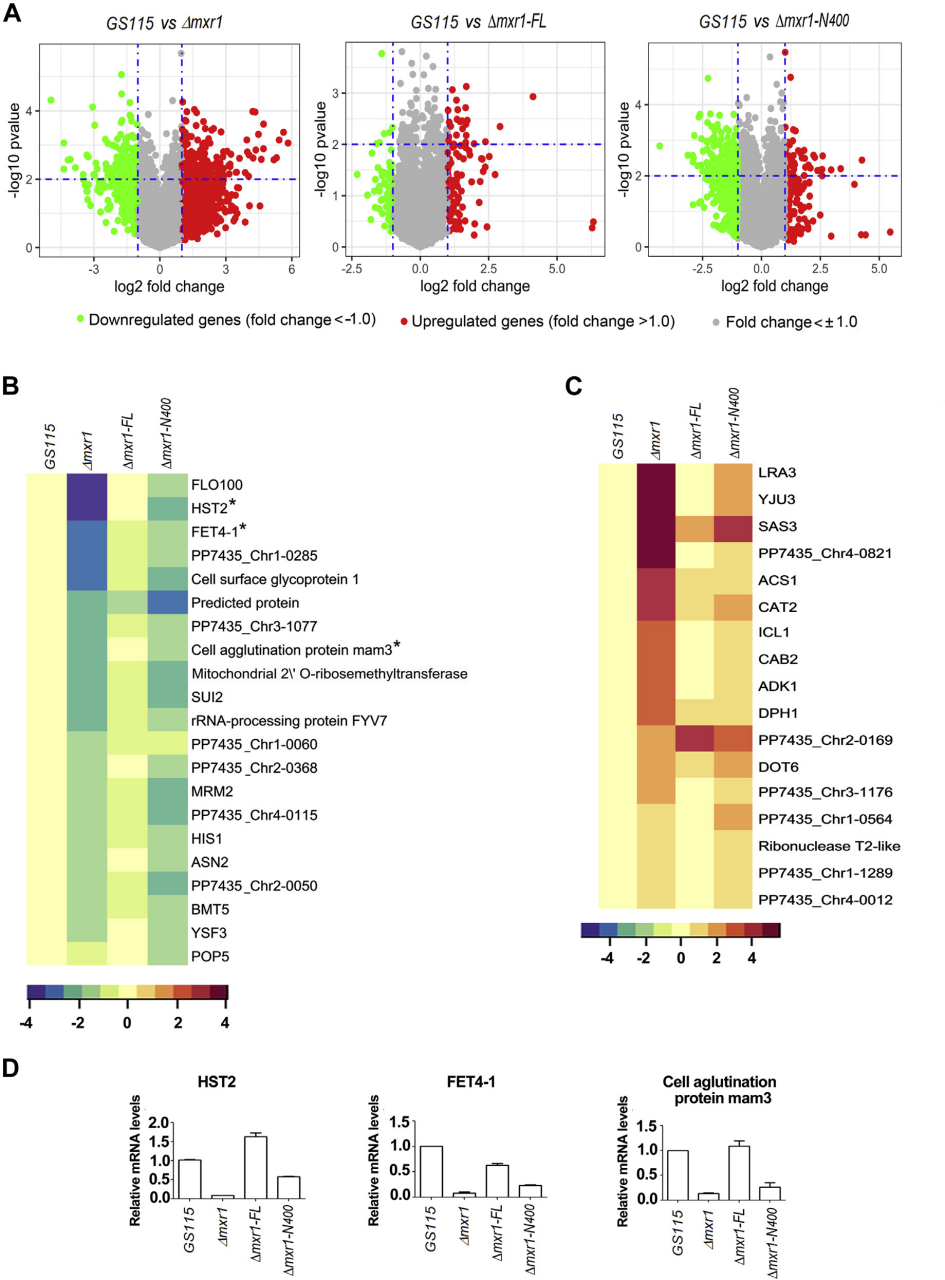
**Identification of methanol metabolism genes differentially regulated by Mxr1N400 and Mxr1FL.***A*, volcano plot highlighting significant upregulated and downregulated genes. Genes differentially expressed between *GS115* and *Δmxr1*, *GS115* and *Δmxr1-FL*, and *GS115* and *Δmxr1-N400* are shown. The *y*-axis corresponds to −log10 of *p* value, and *x*-axis displays the log2 fold change value. *Green* and *red dots* represent downregulated and upregulated transcripts, respectively. *B*, heat map of methanol metabolism genes downregulated in *Δmxr1* relative to *GS115*. When expressed in *Δmxr1*, Mxr1-FL reverses downregulation more effectively than Mxr1N400 (compare *Δmxr1-FL* and *Δmxr1-N400*). *Asterisk* indicates genes whose expression was validated by quantitative PCR (qPCR). *C*, heat map of methanol metabolism genes upregulated in *Δmxr1* relative to *GS115*. When expressed in *Δmxr1*, Mxr1-FL reverses upregulation more effectively than Mxr1N400 (compare *Δmxr1-FL* and *Δmxr1-N400*). To identify significantly regulated genes, a cutoff of *p* value <0.01 was applied. *D*, validation of DNA microarray data by qPCR of select genes. Error bars indicate SD; n = 2.

Comparative analysis of the transcriptome of *GS115*, *Δmxr1*, *Δmxr1-FL*, and *Δmxr1-N400* indicates that Mxr1FL is more efficient than Mxr1N400 in restoring the gene expression pattern of *Δmxr1* to that of *GS115* (Fig. 6, *A*–*C*). At least 21 genes are downregulated in *Δmxr1*, and their expression is restored to almost wildtype levels by Mxr1FL but not Mxr1N400 (Fig. 6*B*) suggesting that the region beyond 400 amino acids of Mxr1p is required for their transactivation. This is further validated by qPCR of select genes such as *HST2* encoding an NAD^+^-dependent protein deacetylase of the silencing information regulator 2 family, *FET4-1* encoding low-affinity Fe (II) transporter of the plasma membrane, and cell agglutination protein mam3 (Fig. 6*D*). *HST2* is one of the most downregulated genes in *Δmxr1*, and its expression is fully restored by the expression of Mxr1FL but not Mxr1N400 (Fig. 6, *B* and *D*). Surprisingly, deletion of Mxr1p resulted in the upregulation of genes as well. A cluster of 17 genes is specifically upregulated in *Δmxr1*, and this is reversed more efficiently by Mxr1FL than Mxr1N400 (Fig. 6*C*). Thus, the region beyond 400 N-terminal amino acids is required for the regulation of full complement of Mxr1p target genes essential for methanol metabolism, and this explains the need for Mxr1FL to rescue the growth defect of *Δmxr1* cultured in YNBM.

**Figure 7:**
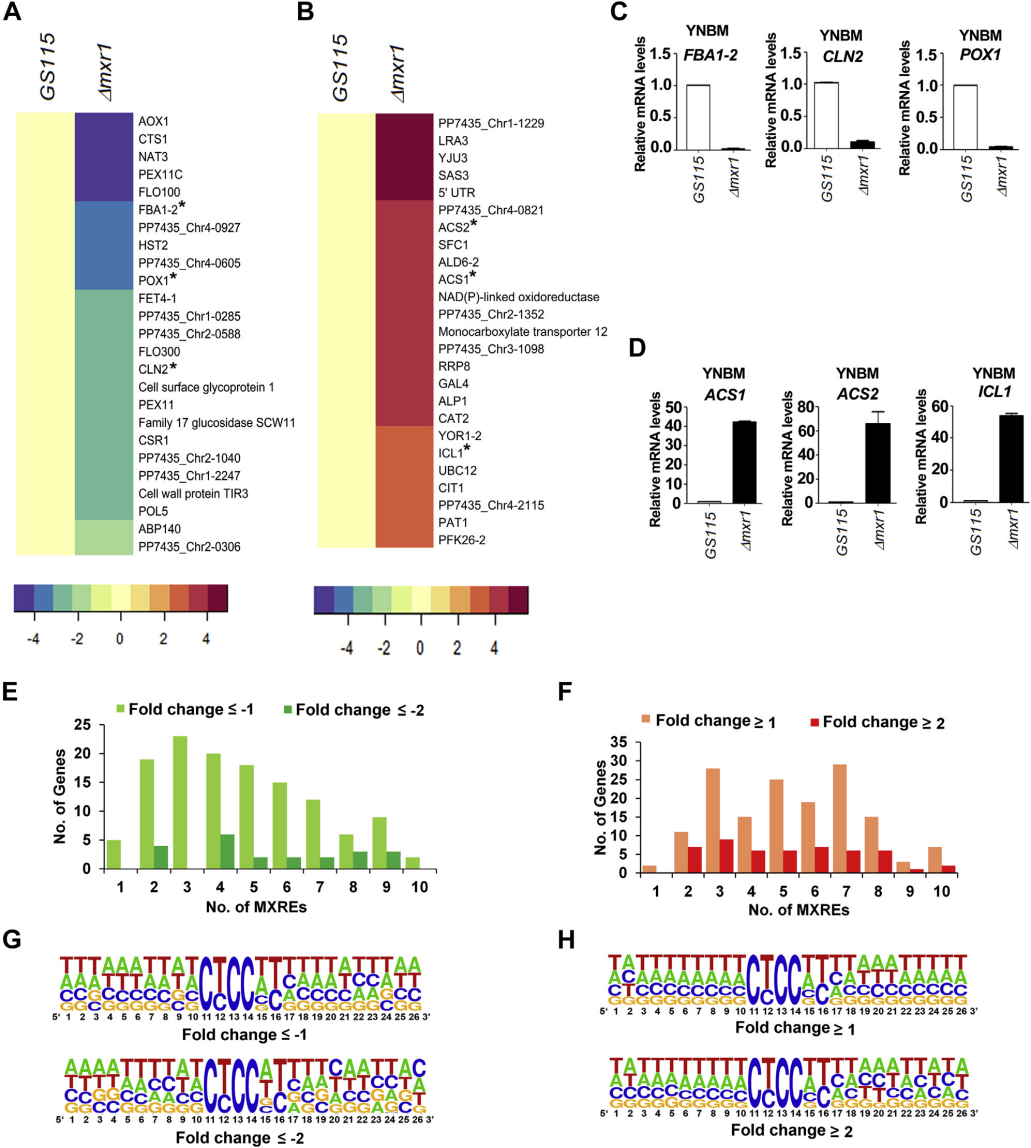
**Novel target genes of Mxr1p involved in methanol metabolism.***A*, 25 most downregulated genes in *Δmxr1* cultured in YNBM. *B*, 25 most upregulated genes in *Δmxr1* cultured in YNBM. *Asterisk* indicates genes whose expression was further validated by quantitative PCR (qPCR). *C*, qPCR validation of select downregulated genes. *D*, qPCR validation of select upregulated genes. Error bars in *C* and *D* indicate SD; n = 2. *E* and *F*, frequency of 5′-CYCCNY-3′ motifs (MXREs) in the promoters (−1.0 kb) of genes downregulated and upregulated in *Δmxr1* cultured in YNBM. *G* and *H*, analysis for the presence of consensus sequences specific for genes downregulated and upregulated in *Δmxr1* cultured in YNBM by MDDLogo (https://weblogo.berkeley.edu/logo.cgi). YNBM, yeast nitrogen base and 1% methanol.

In addition to the demonstration of differential regulation of genes of methanol metabolism by Mxr1FL and Mxr1N400, the DNA microarray analysis has led to the identification of several novel target genes of Mxr1p in cells metabolizing methanol. Deletion of *MXR1* altered the transcriptional landscape drastically (Fig. 6*A*), and a total of 161 genes with fold ≤−1, 82 genes with fold ≤−1.5, and 32 genes with fold ≤−2 were downregulated in *Δmxr1* (Supporting information 3 and Fig. 7*A*). Interestingly, deletion of *MXR1* resulted in upregulation of several genes (Fig. 7*B*), which is surprising, since Mxr1p is known to function as a transcriptional activator rather than transcriptional repressor. In fact, the number of upregulated genes surpassed that of downregulated genes in *Δmxr1*, with roughly 222 genes upregulated ≥1-fold, 135 genes ≥1.5-fold, and 79 genes ≥2-fold (Supporting information 3 and Fig. 7*B*). We have validated the microarray data by qPCR analysis of select genes. These include downregulated genes *FBA1-2*, *CLN2*, and *POX1* encoding fructose-bisphosphate aldolase, G1/S-specific cyclin, and acyl CoA oxidase, as well as upregulated genes *ACS1*, and *ACS2* and *ICL* encoding ACS1 and ACS2 and isocitrate lyase, respectively (Fig. 7, *C* and *D*). The enriched Gene Ontology (GO) categories for the downregulated genes include RNA-related processes, such as RNA methyltransferase, catalytic activity on RNA, metabolic processes, and transferase activity. In case of upregulated genes, enriched GO categories include primarily carboxylic acid, nucleotide, and amino acid metabolic processes (Fig. S8). Mxr1p target genes have been shown to possess the 5′-CYCCNY-3′ consensus sequence (MXRE) in their promoter regions. Therefore, we examined the presence of MXREs within 1.0-kb promoter region of all the differentially regulated genes identified in microarray using Regulatory Sequence Analysis Tools fungi (http://rsat-tagc.univ-mrs.fr/rsat/). Analysis reveals that 82% and 73% of the downregulated and upregulated genes, respectively, harbor atleast one MXRE in their promoter (Supporting informations 4 and 5) indicating that these genes may be direct targets of Mxr1p. Number of genes with 1 to 10 MXREs within 1.0 kb of their promoters are indicated in Figure 7, *E* and *F*. To examine whether MXREs of upregulated genes are different from those of downregulated genes, 5′-CYCCNY-3′ along with flanking sequences were submitted to MDDLogo (https://weblogo.berkeley.edu/logo.cgi). While the 5′-CYCCNY-3′ motif is conserved in both upregulated and downregulated genes, we did not observe enrichment of nucleotides in the flanking sequences (Fig. 7, *G* and *H*) making it difficult to derive a consensus sequence specific for upregulated and downregulated genes.

## Discussion

This study demonstrates for the first time that Mxr1p is required for efficient ethanol metabolism in *P. pastoris* and in its absence, ALD6-1-mediated conversion of acetaldehyde to acetate is impaired resulting in growth defect. This growth defect is not rescued by other ALDs indicating that ALD6-1 has an important and nonredundant function during ethanol metabolism. Mxr1p binds to three MXREs in *ALD6-1* promoter *in vitro*, and a combination of two of them is sufficient for Mxr1p-dependent expression of a reporter gene from *ALD6-1* promoter *in vivo* during ethanol metabolism. This result is similar to that reported in an earlier study in which two MXREs in the promoter of *ACS1* encoding ACS1 were shown to be required for Mxr1p-mediated transactivation during acetate metabolism (4). Mutation of all three MXREs does not completely abrogate GFP expression from *ALD6-1* promoter (Fig. 2*F*) suggesting that transcription factors other than Mxr1p may bind elsewhere in the promoter and contribute to *ALD6-1* promoter activity. Thus far, studies have focused on the Mxr1p-mediated repression of genes of MUT pathway such as *AOX1* during ethanol metabolism (20, 32). Phosphorylation of serine 215 residue of Mxr1p was shown to be involved in the transcriptional repression of *AOX1* during ethanol metabolism (20). Furthermore, acetylation of histones or other transcription factors by acetyl-CoA synthesized during ethanol metabolism may also contribute to the repression of methanol-inducible genes (32). However, no other study except the present study investigated the ability of Mxr1p to regulate the expression of gene(s) essential for ethanol metabolism. While we have focused our attention on *ALD6-1*, the most downregulated Mxr1p target gene in *Δmxr1*, it will be interesting to study other Mxr1p targets as well. Overexpression of *ALD6-1* in *Δmxr1* using a heterologous promoter does not result in restoration of growth (Fig. 1, *M* and *N*) indicating that some of the Mxr1p target genes identified in this study by RNA-Seq may also have a key role during ethanol metabolism.

Study of the transactivation functions of Mxr1p during ethanol metabolism is another important aspect of this study. We demonstrate that Mxr1N400 activates the transcription of not only *ALD6-1* but also all the Mxr1p target genes required for ethanol metabolism as evident from the fact that growth of *Δmxr1* is restored by Mxr1N400 as efficiently as Mxr1FL. We further demonstrate that phenylalanine as well as glutamine residues in Mxr1N400 do not have a role in the transactivation of *ALD6-1* in cells metabolizing ethanol. The region between 251 and 400 N-terminal amino acids of Mxr1p is essential for *ALD6-1* transactivation during ethanol metabolism. Furthermore, 9aaTAD present between 365 and 373 amino acids functions as TAD during ethanol metabolism.

Nuclear localization is essential for Mxr1p to function as a transcription factor. The mechanism by which Mxr1p translocates from cytosol to the nucleus in cells cultured in nonfermentable carbon sources is not known. In this study, we not only identified several putative NLSs using NLS prediction tools but also experimentally validated them and identified one of them (pNLS-C1) as the actual NLS. Whether other NLSs are present in the region beyond 250 N-terminal amino acids remains to be investigated. NLSs enriched in basic amino acids bind to importin α or importin β, and the resultant protein complex is translocated to the nucleus (33). It will be interesting to examine the role of importin α or β in the nuclear import of Mxr1N250. NLSs are not well characterized in *P. pastoris* (34), and this is first reported on the detailed characterization of NLS of a *P. pastoris* transcription factor.

Study of transactivation functions of Mxr1p during methanol metabolism led to interesting results. Phenylalanine residues (F249, F254, and F278) that had previously been reported to be indispensable for transactivation function of Mxr1p by Parua *et al.* (20) were found to have no role in the activation of either *ALD6-1* or *AOX1* during ethanol and methanol metabolism, respectively. This discrepancy is primarily because of different experimental systems used in these two studies. It should be noted that Parua *et al.* (20) examined transactivation by Mxr1p TAD (246–280 amino acids) fused to Gal4-DBD of *lacZ* gene from a GAL4-responsive promoter in *S. cerevisiae* metabolizing glucose (20). Our study is physiologically more relevant as we have investigated the role of phenylalanine and glutamine residues of Mxr1N400 in the transactivation of native Mxr1p target genes such as *ALD6-1* and *AOX1* in *P. pastoris* rather than a heterologous yeast such as *S. cerevisiae*. Despite mutation of phenylalanine and glutamine residues, Mxr1N400 still activated *AOX1* transcription indicating that other amino acid residues contribute to the transactivation function of Mxr1N400. In fact, Mxr1N250 failed to activate *AOX1* transcription during methanol metabolism as well (data not shown) suggesting that the region between 251 and 400 amino acids may also be involved in the activation of genes of methanol metabolism.

The fact that Mxr1N400 failed to rescue the growth defect of *Δmxr1* during the methanol metabolism despite activating *AOX1* as well as other genes known to be involved in MUT pathway suggested that the region beyond 400 N-terminal amino acids is essential for the regulation of other Mxr1p target genes required for methanol metabolism. DNA microarray analysis clearly demonstrated that Mxr1FL restores the expression of several genes downregulated in *Δmxr1* more efficiently than Mxr1N400. This is true for genes upregulated in *Δmxr1* as well. It will be interesting to examine the role of 9aaTAD identified in this study as well as the putative 9aaTADs present between 401 and 1155 amino acids in the regulation of Mxr1p target genes during methanol metabolism.

This study demonstrates that Mxr1FL regulates the expression of several essential genes for methanol metabolism in addition to the previously reported classical MUT pathway genes. For example, the expression of several genes involved in epigenetic regulation such as histone acetyltransferases and histone deacetylases are regulated by Mxr1p (Supporting information 3 and Fig. 6, *B* and *C*). In fact, HST2 encoding a histone deacetylase of the silencing information regulator 2 family is one of the most downregulated genes in *Δmxr1* during methanol metabolism. Investigation of the role of HST2 in methanol metabolism is a topic for future research. The upregulation of several genes in *Δmxr1* during ethanol as well as methanol metabolism suggests that Mxr1p may function as a transcriptional repressor as well. In fact, 73% of genes upregulated in *Δmxr1* cultured in YNBM possess at least one MXRE in their promoters. While the core MXRE (5′–CYCCNY-3′) was present in the promoters of both upregulated and downregulated genes, sequences flanking the core MXRE did not exhibit high degree of conservation rendering it difficult to derive a consensus sequence specific for upregulated and downregulated genes. Functional characterization of MXREs of genes upregulated in *Δmxr1* is a topic of future research. In addition to the identification of Mxr1p as a key regulator of ethanol metabolism, this study provides new insights into the mechanism by which Mxr1p functions as a global regulator of multiple metabolic pathways of *P. pastoris*.

## Experimental procedures

### Growth media and culture conditions

*P. pastoris* cells were maintained in nutrient-rich yeast extract, peptone, and dextrose (YPD) agarose plates (1% yeast extract, 2% peptone, and 2% dextrose) and a single colony was cultured overnight in YPD at 30 °C in an orbital shaker at 180 rpm followed by washing with sterile distilled water (twice) and transferred to desired media consisting of 0.67% YNB (YNB without amino acids and with ammonium sulphate) supplemented with a carbon source 1% methanol (YNBM) or 1% ethanol (YNBE). Yeast extract and peptone were purchased from Becton and Dickinson Biosciences, India. *E. coli TOP10* was used for plasmid isolation while expression of recombinant proteins was carried out in *BL21(DE3)* strain of *E. coli*. Bacterial and yeast transformations were done using the CaCl_2_ method and electroporation (Gene Pulsar; Bio-Rad), respectively.

### Antibodies and other reagents

Anti–phosphoglycerate kinase (PGK) polyclonal antibody was generated by immunizing the rabbit with a purified 6× His-tagged *P. pastoris* PGK protein. Mouse anti-Myc was purchased from Merck Millipore. Restriction enzymes and T4 DNA ligase were purchased from New England Biolabs. DNA polymerases were purchased from GeNei. Oligonucleotides were purchased from Sigma–Aldrich. Nucleotide sequence of primers used in qPCR and RT-PCR will be provided on request.

### Growth kinetics

A single colony was inoculated in 5 ml YPD and grown at 30 °C in an orbital shaker at 180 rpm for 18 to 24 h or till late log phase. Cells were pelleted and washed twice with autoclaved milliQ under sterile conditions and resuspended in water. Cells were then inoculated into specific media at an initial absorbance of 0.06 to 0.1 at 600 nm and grown at 30 °C at 180 rpm in an orbital shaker. Absorbance at 600 nm was measured at regular intervals till cells attain stationary phase.

### Subcellular localization studies

Localization of N-terminal GFP-tagged Mxr1 strains was performed by direct fluorescence. For this, cells cultured in desired medium for 14 to 16 h were fixed by incubating with 3.7% formaldehyde in an orbital shaker at 180 rpm at 30 °C for 1 h. Cells were then harvested, washed with 1× PBS (137 mM NaCl, 2.68 mM KCl, 10 mM Na_2_HPO_4_, 1.8 mM KH_2_PO_4_), followed by spheroplast preparation by incubating with 5 μl of Longlife Zymolyase (G-Biosciences; 1.5 U/ml) in 200 μl of zymolyase buffer (1.2 M sorbitol, 40 mM phosphate buffer, 50 mM MgCl_2_, and 28.4 mM β-mercaptoethanol) at 37 °C for 30 min. Harvested spheroplasts were washed and resuspended in 1× PBS + 0.05% Tween-20. Smears were prepared and fixed by treatment with methanol and acetone. For nuclear staining, fixed smears were incubated with 1 mg/ml of Hoechst 33342 in 1× PBS for 7 min in dark, followed by washing with 1× PBS for 10 min, and mounting on slide and sealing with nail polish. Slides were visualized in confocal microscope (Olympus Fluoview FV3000) with a 100× objective.

### Real-time qPCR

*P. pastoris* cells were cultured for 12 to 14 h, and total RNA was isolated using RNA isolation kit (catalog no. Z3100; Promega) as per the manufacturer's instructions. RNA was quantified by measuring the absorbance at 260 nm using NanoDrop 2000 spectrophotometers, and 1 μg of DNase-treated RNA was used for complementary DNA (cDNA) preparation. qPCR was carried out using iQ SYBR Green super mix in a StepOnePlus Real-Time PCR system (Thermo Fisher Scientific). The relative mRNA expression levels were obtained using the ΔΔCt method, wherein the ΔCt value of a sample, which is the Ct value of a gene relative to that of 18S rRNA, is normalized to that of the control.

### Western blotting

Cells were lysed using glass-bead lysis method followed by quantification by Bradford assay, separation of protein bands on SDS-PAGE, transfer to polyvinylidene fluoride membrane, and probing with primary and secondary antibodies. ImageJ software (https://imagej.nih.gov/ij/) was applied to quantify all Western blot data. Band intensity of the protein of interest was normalized to that of the loading control, PGK.

### Statistical analysis

Student's *t* test was carried out using GraphPad Prism 5, software (GraphPad Software, Inc). Data are represented as mean ± SD. *p* Value summary is mentioned on the bar of each figure where ∗*p* < 0.05; ∗∗*p* < 0.005; ∗∗∗*p* < 0.0005, and ns, not significant.

### RNA-Seq and data analysis

Total RNA was isolated from *GS115* and *Δmxr1* cultured in duplicates in YNBE for 14 h by Qiagen RNeasy kit according to the manufacturer's protocol. RNA-Seq was performed using Illumina HiSeq. FastQC and MultiQC software were used to check data quality. The data were checked for sequencing adapter contamination, base call quality distribution, percent of bases above Q20, Q30, and %GC. For all the samples, quality control was above threshold (Q20 > 95%). Fastp was used to process raw sequence reads by removal of adapter sequences and low-quality bases. Bowtie2 aligner was used to align the quality control–passed reads onto indexed *K. phaffii* CBS 7435 reference genome (GCA_900235035.1_ASM90023503v1). About 94.73% of the reads could be mapped to the reference genome on average. Gene level expression values were obtained as read counts using feature counts software. Expression similarity between the biological replicates was checked by Spearman correlation and principal component analysis. DESeq2 package was used for differential expression analysis. Genes with less than five reads in any of the two samples were removed from further analysis. The read counts were normalized (variance stabilized normalized counts) using DESeq2, and differential enrichment analysis was performed. The protein sequences were annotated against the Uniref100 protein database using BLASTp module of Diamond. The gene names, length, GO annotations, and other additional information were obtained by submitting the Uniref100 Ids to UniProtKB. Genes with absolute log2 fold change ≥1 and adjusted *p* value ≤0.05 were considered significant. The expression profile of differentially expressed genes across the samples is presented in volcano plot and heat map. The enriched GO categories were retrieved from Amigo using *S. cerevisiae* as organism. The transcriptome datasets generated during the current study are available in the National Center for Biotechnology Information (NCBI) with the accession number GSE168677.

### Microarray hybridization and data analysis

For the generation of samples for microarray analysis, *GS115*, *Δmxr1*, *Δmxr1-FL*, and *Δmxr1-N400* were cultivated in biological duplicates in YNBM for 14 h. Total RNA extraction was performed using Qiagen RNeasy kit according to the manufacturer's protocol. The microarray hybridization and scanning were performed at the Agilent-certified microarray facility of Genotypic Technology, Bengaluru, India. The samples for gene expression were labeled using Agilent Quick-Amp Labeling Kit (part number: 5190-0442). The total RNA was reverse transcribed at 40 °C using oligodT primer tagged to a T7 polymerase promoter and converted to double-stranded cDNA. Synthesized double-stranded cDNA was used as a template for cRNA generation by *in vitro* transcription, and the dye Cy3 CTP (Agilent) was incorporated during this step. The cDNA synthesis and *in vitro* transcription steps were carried out at 40 °C. Labeled cRNA was cleaned up using Qiagen RNeasy columns (Qiagen; catalog no.: 74106), and quality was assessed for yields and specific activity using the Nanodrop ND-1000. Labeled cRNA sample was fragmented at 60 °C and hybridized on to Agilent *P. pastoris* Gene Expression Microarray 8X15K AMADID: 085736-One Color Platform. Fragmentation of labeled cRNA and hybridization were done using the Gene Expression Hybridization Kit of (*In situ* Hybridization kit, part number: 5190-0404; Agilent Technologies). Hybridization was carried out in Agilent's SureHyb Chambers at 65 °C for 16 h. The hybridized slides were washed using Agilent Gene Expression wash buffers (Agilent Technologies; part number 5188-5327) and scanned using the Agilent Microarray Scanner (part number G2600D; Agilent Technologies). Raw data extraction from images was obtained using Agilent Feature Extraction software. Feature-extracted raw data were analyzed using Agilent GeneSpring GX (version 14.5) software. Normalization of the data was done in GeneSpring GX using the 75th percentile shift method. Significant genes upregulated with fold change ≥1 (logbase2) and downregulated with fold change ≤−1 (logbase2) in the test samples with respect to control sample were identified. Statistical Student's *t* test *p* value among the replicates was calculated based on volcano plot algorithm. Differentially regulated genes were clustered using hierarchical clustering based on the Pearson's coefficient correlation algorithm to identify significant gene expression patterns. Data were analyzed as described in RNA-Seq section. The raw data files are available at the NCBI with an accession number GSE146829.

### Generation of *Δmxr1*, *Δmxr1-FL*, and *Δmxr1-N400*

*Δmxr1* was generated by disrupting the *MXR1* coding region by zeocin resistance cassette (*Zeo*^*R*^) in *GS115* as has been previously described (4). *Δmxr1-FL* and *Δmxr1-N400* were generated by transforming plasmid expressing *MXR1FL* and *MXR1N400* as C-terminal Myc-tagged proteins under GAPDH promoter as described (4).

### Generation of *Δald6-1*

A 0.911-kb *ALD6-1* promoter region was amplified by PCR from *P. pastoris* genomic DNA using primer pair 5′-GGACTGTTCAATTTGAAGTCGATGCTGACG-3′ and 5′-GCTATGGTGTGTGGGGGATCC GCACACGATCCCTTGGGAACTTGCGGTGG-3′ (962–984 bp of *GAPDH* promoter in reverse complement, −89 to −114 bp of *ALD6-1* promoter in reverse complement). In another PCR, 1.2 kb of zeocin expression cassette was amplified by PCR from *pGAPZA* vector using the primer pair 5′-CCACCGCAAGTTCCCAAGGGATCGTGTGCGGATCCCCCACACACCATAGC-3′ (−89 to −114 bp of *ALD6-1* promoter, +962 to +984 bp of *GAPDH* promoter) and 5′GGAGTGTAAG CAATTCTGATAGCCTTGTGCCACATGTTGGTCTCCAGCTTG-3′ (+1467 bp to +1493 bp in reverse complement of 3′-flanking region of *ALD6-1*, +2137 to +2159 bp in reverse complement of *GAPDH* promoter). In the third PCR, 872 bp of the 3′-flanking region of *ALD6-1* was amplified using the primer pair 5′-CAAGCTGGAGA CCAACATGTGAGCACAGGCTATCAGAATTGCTTACACTCC-3′ (+2137 to +2159 bp of *GAPDH* promoter, +1467 to +1493 bp of region of *ALD6-1* gene) and 5′-GGAACTGGAG GCTTCCGCAGCAAACTCTC-3′ (+2352 to +2381 bp in the reverse complement of 3′-flanking region of *ALD6-1*). All the three PCR products were purified and used as a template in the final PCR and amplified using a primer pair 5′-GGACTGTTCAATTTGAAGTCGATGCTGACG-3′ and 5′-GGAACTGGAGGCTTCCGCAGC AAACTCTC-3′ to obtain a 3.023-kb product consisting of zeocin expression cassette along with promoter and terminator of *ALD6-1*. *GS115* strain was transformed with *Zeo*^*R*^ expression cassette, and zeocin-resistant colonies were selected. Deletion of *ALD6-1* was confirmed by PCR using gene-specific primers.

### Generation of *GS115-A*, *Δmxr1-A*, and *Δmxr1-A-N400*

The gene encoding ALD6-1 along with 1.0 kb of its promoter was cloned into *pIB3* vector (#25452; Addgene) and transformed into *GS115*, *Δmxr1*, and *Δmxr1-N400*. The following primer pair was used: 5′-TCCCCCCGGGATTGGAGAAGACAATGAATCTG-3′ and 5′-CCGCTCGAGCTACAGGTCTTCTTCAGAGTCAGTTTCTGTTCCTTATGTCAGGAGTGTAAGC-3′ (XmaI and XhoI sites in the primers are underlined). The reverse primer encoded the Myc tag. The PCR product was cloned into *pIB3* vector, the recombinant plasmid was linearized using SalI, and transformed into *GS115*, *Δmxr1*, and *Δmxr1-N400*. Recombinant clones were selected by plating on YNBD His^−^ plates, and clones expressing Myc-tagged ALD6-1 (ALD6-1^Myc^) were identified by Western blotting using anti-Myc epitope antibody.

### Generation of *GS115-P*_*A*_*-GFP* and *Δmxr1-P*_*A*_*-GFP*

These strains express GFP from 1.0 kb of *ALD6-1* promoter and were constructed as described later. Three different PCRs were carried out for the construction of *P*_*A*_*GFP* expression cassette. In the first PCR, 1 kb *ALD6-1* promoter was amplified from genomic DNA of *GS115* strain of *P. pastoris* using a primer pair 1F (5′-CGGGATCCATTGGAGAAGACAATGAATCTGAC-3′ [BamHI site is underlined]) and 1R (5′-CTCCTTTACTAGTCAGATCTACCATGGATAAAGGTAAGGGAAAAAAGCA AGTG-3′; +1 to +25 bp of the gene encoding GFP and −1 to −28 bp of *P*_*A*_). In the second PCR, a 714-bp region of *GFP* gene was amplified from pREP41GFP vector using the primer pair 2F (5′-CACTTGCTTTTTTCCCTTACCTTTATCCATGGTAGATCTGACTAGTAAAGGAG-3′, −1 to −28 bp of *P*_*A*_) and +1 to +25 bp of the gene encoding GFP and 2R (5′-CCGCTCGAGCTAGTGGTGGTGGCTAGCT TTG-3′). The XhoI site is underlined. In the third PCR, the PCR products from the first two reactions were used as templates and amplified using the 1F and 2R primers to get the *P*_*A*_*-GFP* expression cassette, which was digested with BamHI and XhoI and cloned into pIB3 (catalog no. 25452; Addgene) to generate *pIB3-P*_*A*_*-GFP*. The generated expression vectors were linearized with SalI and transformed into *GS115* and *Δmxr1* strains and plated on YNBD-His^−^ agar plates, and positive colonies were screened using anti-GFP antibody.

### Generation of *GS115-P*_*A-M1*_*-GFP*, *GS115-P*_*A-M2*_*-GFP*, *GS115-P*_*A-M3*_*-GFP*, and *GS115-P*_*A-M4*_*-GFP*

*GS115* expressing GFP from 1.0-kb *ALD6-1* promoter in which each of the three MXREs (M1, M2, and M3), two MXREs, and all the three MXRES (M4) are mutated were generated as follows: *P*_*ALD6-1*_*GFP* plasmid with MXRE-M1 was generated as follows: *ALD6-1* promoter was PCR amplified using primer pair 1F (5′-CGGGATCCATTGGAGAAGACAATGAA TCTGAC-3′ [BamHI site is underlined]) and 1R (5′-CCCTCTCCAAAAGAGGGACGATGTAGATGAGAACCGTTGAGCGGAATCATG GGTG-3′). In another PCR, *GFP* was amplified using primer pair 2F (5′-CACCCATGATTC CGCTCAACGGTTCTCATCTACATCGTCCCTCTTTTGGAGAGGG-3′ [mutation is underlined]) and 2R (5′-CCGCTCGAGCTAGTGGTGGTG GCTAGCTTTG-3′ [the XhoI site is underlined]). In the third PCR, the PCR products from the first two reactions were used as templates and amplified using the 1F and 2R primers. Thereafter, the amplicon was digested with BamHI and XhoI and cloned into *pIB3* (Addgene) to generate a plasmid carrying mutation in MXRE1 (*pP*_*A*_*-GFP-M1*). Similar strategy was used to generate the plasmids, *pP*_*A*_*-GFP-M2 pP*_*A*_*-GFP-M3*, and *pP*_*A*_*-GFP-M4* carrying mutations in MXRE2 alone, MXRE3 alone, or all three MXREs, respectively, using appropriate 1R mutant reverse primers. Plasmids were linearized with SalI and transformed into *GS115* to obtain the strains *GS115-P*_*A-M1*_*GFP*, *GS115-P*_*A-M2*_*GFP*, *GS115-P*_*A-M3*_*GFP*, and *GS115-P*_*A-M4*_*GFP*.

### Generation of *Δmxr1-A-N400F∗*, *Δmxr1-A-N400Q∗*, and *Δmxr1-A-N400F∗Q∗*

To introduce mutations in *Mxr1N400*, codon-optimized *MXR1N400* genes were synthesized. *MXR1N400F∗* was synthesized at TWIST Biosciences, wherein the phenylalanine residues at 249, 254, and 278 positions were mutated to alanine residues in *MXR1N400* gene. In the *MXR1N400* gene, 18 glutamine residues between 150 and 200 amino acids (152, 155, 157, 158, 159, 162, 163, 164, 166, 168, 170, 171, 175, 179, 181, 182, 185, and 194) were mutated to alanine to get *MXR1N400Q∗*. This gene was synthesized by GeneArt (Thermo Fisher Scientific). Another synthetic gene, *MXR1N400F∗Q∗*, with phenylalanine residues at 249, 254, and 278 mutated to alanine, and 18 glutamine residues between 150 and 200 amino acids (152, 155, 157, 158, 159, 162, 163, 164, 166, 168, 170, 171, 175, 179, 181, 182, 185, and 194) mutated to alanine in the *MXR1N400* were synthesized at TWIST Biosciences. All the three synthetic genes were cloned in *pGHYB* (35) vector with an N-terminal *GFP* tag under *GAPDH* promoter, with subsequent transformation in *Δmxr1-A* and plating on Hyg^r^ plates. Screening for positive colonies was done by Western blotting and probing with anti-GFP antibody. Sequence of the synthetic gene will be made available upon request.

### Generation of *Δmxr1-N400ΔTAD*

To delete a putative 9aaTAD (365–373), two Mxr1 fragments were amplified, which were then fused by overlapping PCR. In the first set of PCR, *MXR1N365* with an overhang of 15 to 20 bases downstream of *MXR1N373* was amplified with a forward primer having a KpnI site followed by 1 to 26 bases of *MXR1* sequence 5′-CGGGGTACCATGAGCAATCTACCCCCAACTTTTGG-3′ (F1) and reverse primer is reverse complement of 21 bases upstream of *MXR1N363* followed by 20 bases downstream of *MXR1N373* 5′-GGCAAGCAAAAATTCTTGGAAATGGTATTAGCAGCCGGTGC-3′ (R1). In the second set of PCR, *MXR1N373-400* region was amplified with a 5′ overhang of bases upstream of *MXR1N365* with a forward primer having 21 bases upstream of *MXR1N363* followed by 20 bases downstream of *MXR1N373* 5′-GCACCGGCTGCTAATACCATTTCCAAGAATTTTTGCTTGCC-3′ (F2) and reverse primer is reverse complement of sequence of *MXR1* 1174 to 1200 followed by Myc-tag, stop codon, and NotI restriction enzyme site 5′-TTTTCCTTTTGCGGCCGCCTACAGATCCTCTTCTGAGATGAGTTTTTGTTCGCATGATAAC GTGTTAGAGAAAGTCTG-3′ (R2). The two fragments generated by the first and second PCRs—*MXR1N365* and *MXR1N373–400*—were used as template for the overlapping PCR with forward primer F1 and reverse primer R2. This fused PCR product was digested with KpnI and NotI restriction enzymes and cloned under *GAPDH* promoter in *pGHYB* (35) vector, followed by transformation in *Δmxr1*. Positive colonies were screened for expression of Myc-tagged Mxr1N400ΔTAD using anti-Myc epitope antibody.

### Generation of *Δmxr1-A-N150*, *Δmxr1-N250*, and *Δmxr1-A-N250*

To construct an N-terminal *GFP*-tagged *Mxr1N250* and N-terminal *GFP*-tagged *Mxr1N150*, first, the GFP sequence was amplified from *pEGFP-C2* (catalog no. 6083-1; Addgene) with the forward primer having N-terminal region (1–18 base pairs) of the *GFP* gene with an upstream KpnI restriction enzyme site 5′-CGGGGTACCATGGTGAGCAAGGGCGAG-3′ (F) and the reverse primer complementary to C-terminal region (1311–1329 base pairs) of *GFP* and overlapping sequence with the N-terminal region of (1–26 base pairs) *MXR1* gene 5′-CCAAAAGTTGGGGGTAGATTGCTCATCTTGTACAG CTCGTCCATG-3′. In another set of PCRs, Mxr1p sequence was amplified using a common forward primer for amplification of first 150 or 250 bp of *MXR1*, which have an overlapping sequence of C-terminal region (1311–1329 base pairs) of *GFP* gene followed by N-terminal region (1–26 base pairs) of *MXR1* gene 5′-CATGGACGAGCTGTACAAGATGAGCAATCTACCCCCAACTTTTGG-3′ (F′). The reverse primer for amplification of *MXR1N150* is sequence reverse complementary to 432 to 450 bp of *MXR1* gene with a downstream Myc tag sequence, stop codon, and a NotI restriction enzyme site 5′-ATAGTTTAGCGGCCGCCTACTCAAATTCGGCATTATTTGAATC-3′ (R1). For amplifying *MXR1N250*, the sequence of reverse primer is reverse complementary to 727 to 750 bp of *MXR1* gene with a downstream Myc-tag sequence, stop codon, and a NotI restriction enzyme site 5-ATAGTTTAGCGGCCGCCTACTCAAATTCGGCATTATTTGAATC-3′ (R2). To get the GFP–MXR fusion product, an overlapping PCR was performed where the amplified *GFP* and *MXR1N150* or *MXR1N250* PCR products were used as templates, and primer pairs F and R1 or F and R2 were used. This fused PCR product was digested with KpnI and NotI restriction enzymes and cloned under *GAPDH* promoter in *pGHYB* (35) vector, followed by transformation of *MXR1N250* in *Δmxr1* and *Δmxr1-A* while *MXR1N150* in *Δmxr1-A*. Positive colonies were screened for expression of GFP-tagged Mxr1N250 or Mxr1N150 using anti-GFP antibody.

### Generation of *Δmxr1-N62*, *Δmxr1-N81*, and *Δmxr1-N109*

To construct an N-terminal *GFP*-tagged *Mxr1N62*, N-terminal *GFP*-tagged *Mxr1N81*, and N-terminal *GFP*-tagged *Mxr1N109*, first the GFP sequence was amplified from *pEGFP-C2* (catalog no. 6083-1; Addgene) with the forward primer having N-terminal region (1–18 base pairs) of the *GFP* gene with an upstream KpnI restriction enzyme site 5-CGGGGTACCATGGTGAGCAAGGGCGAG-3′ (F) and the reverse primer complementary to C-terminal region (1311–1329 base pairs) of *GFP* and overlapping sequence with the N-terminal region of (1–26 base pairs) *MXR1* gene 5-CCAAAAGTTGGGGGTAGATTGCTCATCTTGTACAG CTCGTCCATG-3′. In another set of PCRs, Mxr1p sequence was amplified using a common forward primer for amplification of first 62, 81, or 109 bp of *MXR1*, which have an overlapping sequence of C-terminal region (1311–1329 base pairs) of *GFP* gene followed by N-terminal region (1–26 base pairs) of *MXR1* gene 5′-CATGGACGAGCTGTACAAGATGAGCAATCTACCCCCAACTTTTGG-3′ (F′). The reverse primer for amplification of *MXR1N62* is sequence reverse complementary to 169 to 186 bp of *MXR1* gene with a downstream, stop codon, and a NotI restriction enzyme site 5′-ATAGTTTAGCGGCCGCCTAGTGAGACCTTTCGTGTCG-3′ (R1). For amplifying *MXR1N81*, the sequence of reverse primer is reverse complementary to 223 to 243 bp of *MXR1* gene with a downstream stop codon, and a NotI restriction enzyme site 5′-ATAGTTTAGCGGCCGCCTAATCTCGACGGCTGAATTTACG-3′ (R2). For amplifying *MXR1N109*, the sequence of reverse primer is reverse complementary to 312 to 327 bp of *MXR1* gene with a downstream stop codon, and a NotI restriction enzyme site 5′-ATAGTTTAGCGGCCGCCTACCGACGAGTTGCCTTG-3′ (R3). To get the GFP–MXR fusion product, an overlapping PCR was performed where the amplified *GFP* and *MXR1N62*, *MXR1N81*, or *MXR1N109* PCR products were used as templates, and primer pairs F and R1, F and R2, or F and R3 were used. This fused PCR product was digested with KpnI and NotI restriction enzymes and cloned under *GAPDH* promoter in *pGHYB* (35) vector, followed by transformation of *MXR1N62*, *MXR1N81*, and *MXR1N109*, in *Δmxr1*. Positive colonies were screened for expression of GFP-tagged Mxr1N62, Mxr1N81, or Mxr1N109 using anti-GFP antibody.

### Generation of *Δmxr1-N250-M1* and *Δmxr1-N250-M2*

Mutations were introduced in the *MXR1N250* gene by synthesizing codon-optimized genes. *MXR1N250-M1* was generated by mutating the arginine residues at 101, 103, 104, 108, and 109 to alanine residues. *MXR1N250-M2* had substitution mutations at 75, 79, 80, 85, 101, 103, 104, 108, and 109 positions. Both were synthesized at TWIST Biosciences. Synthetic genes were cloned in *pGHYB* (35) with an N-terminal *GFP* tag under *GAPDH* promoter followed by transformation in *Δmxr1* and screening by Western blotting with anti-GFP antibody. Sequence of synthetic genes will be made available upon request.

## Data availability

The RNA-Seq and DNA microarray data are available at NCBI GEO (https://www.ncbi.nlm.nih.gov/geo/) under the accession numbers GSE168677 and GSE146829, respectively.

## Supporting information

This article contains supporting information.

## Conflict of interest

The authors declare that they have no conflicts of interest with the contents of this article.
