# Supplementary material for: Characterization of the transactivation and nuclear localization functions of Pichia pastoris zinc finger transcription factor Mxr1p
Source: J Biol Chem. 2021 Sep 25;297(4):101247. doi: 10.1016/j.jbc.2021.101247 (PMC8526985; doi:10.1016/j.jbc.2021.101247)
Supplement: Supplemental Figures S1–S8 [file mmc1.pdf]

Characterization of the transactivation and nuclear localization functions of *Pichia pastoris* zinc finger transcription factor Mxr1p

Aditi Gupta, Kamisetty Krishna Rao, Umakant Sahu and Pundi N Rangarajan*
Department of Biochemistry, Indian Institute of Science, Bangalore 560012, INDIA

*To whom correspondence should be addressed: Pundi Rangarajan, Department of Biochemistry, Indian Institute of Science, Bangalore 560012, INDIA. Email: pnr@iisc.ac.in Tel: 91 80 22932540

Running title: *Key functional domains of Pichia pastoris Mxr1p*

Key words: yeast metabolism, *Pichia pastoris,* Mxr1p, transcriptional regulation, aldehyde dehydrogenase, alcohol oxidase, transactivation domain, nuclear localization

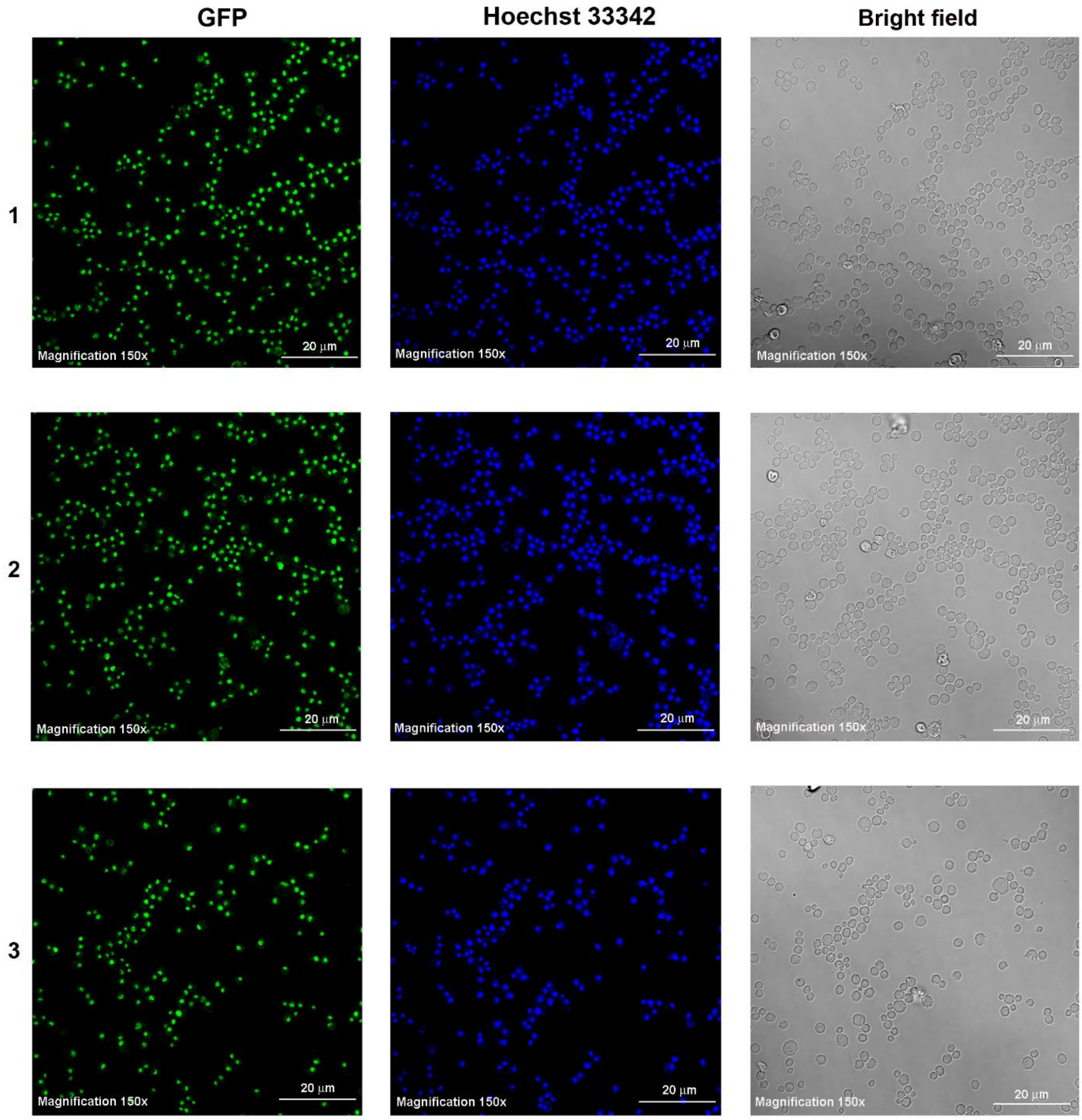

Fig. S1. Subcelular localization of Mxr1N250. Images (GFP, Hoechst 33342, bright field) of three biological replicates (1-3) are shown.

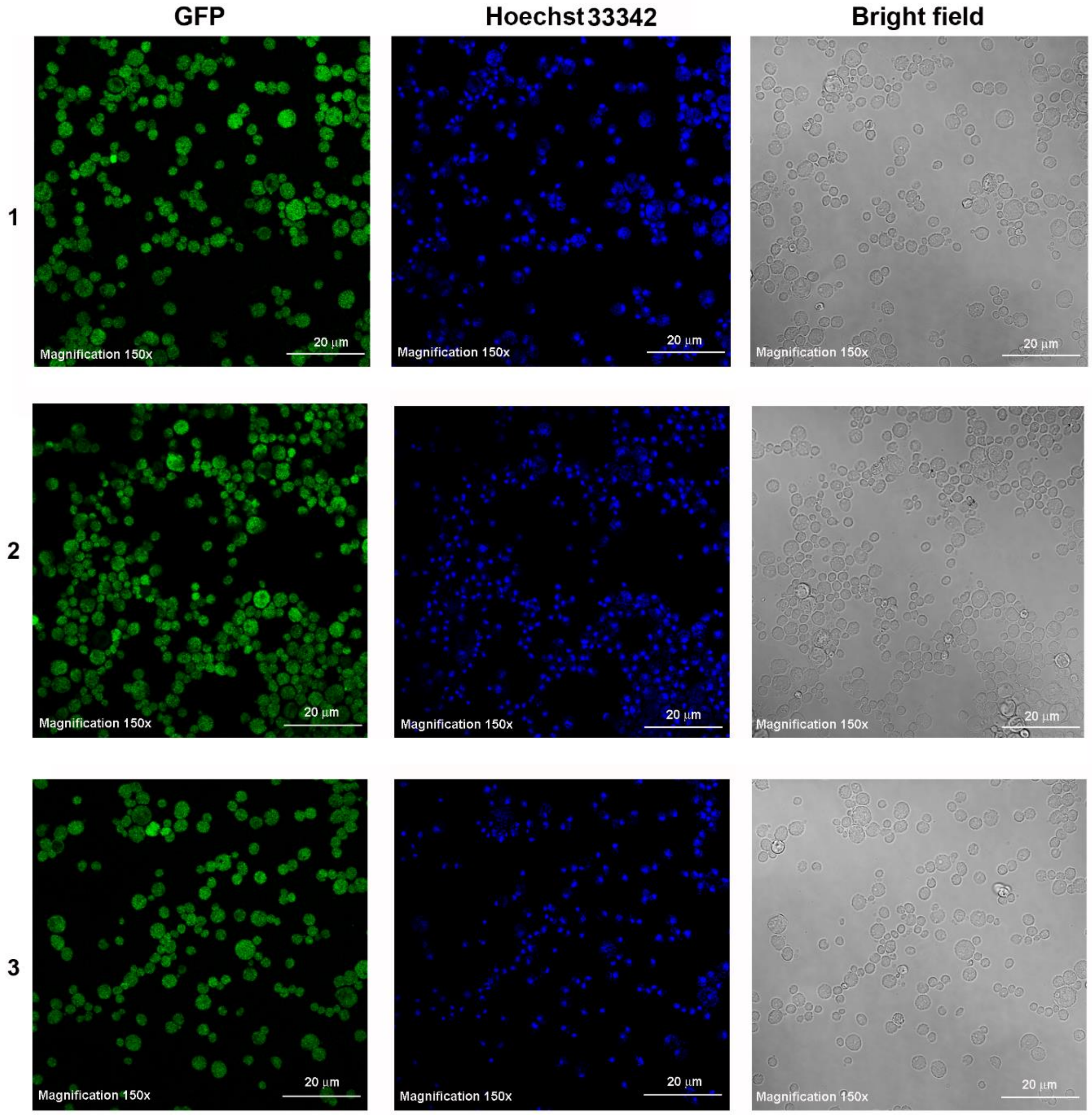

Fig. S2. Subcelular localization of Mxr1N62. Images (GFP, Hoechst 33342, bright field) of three biological replicates (1-3) are shown.

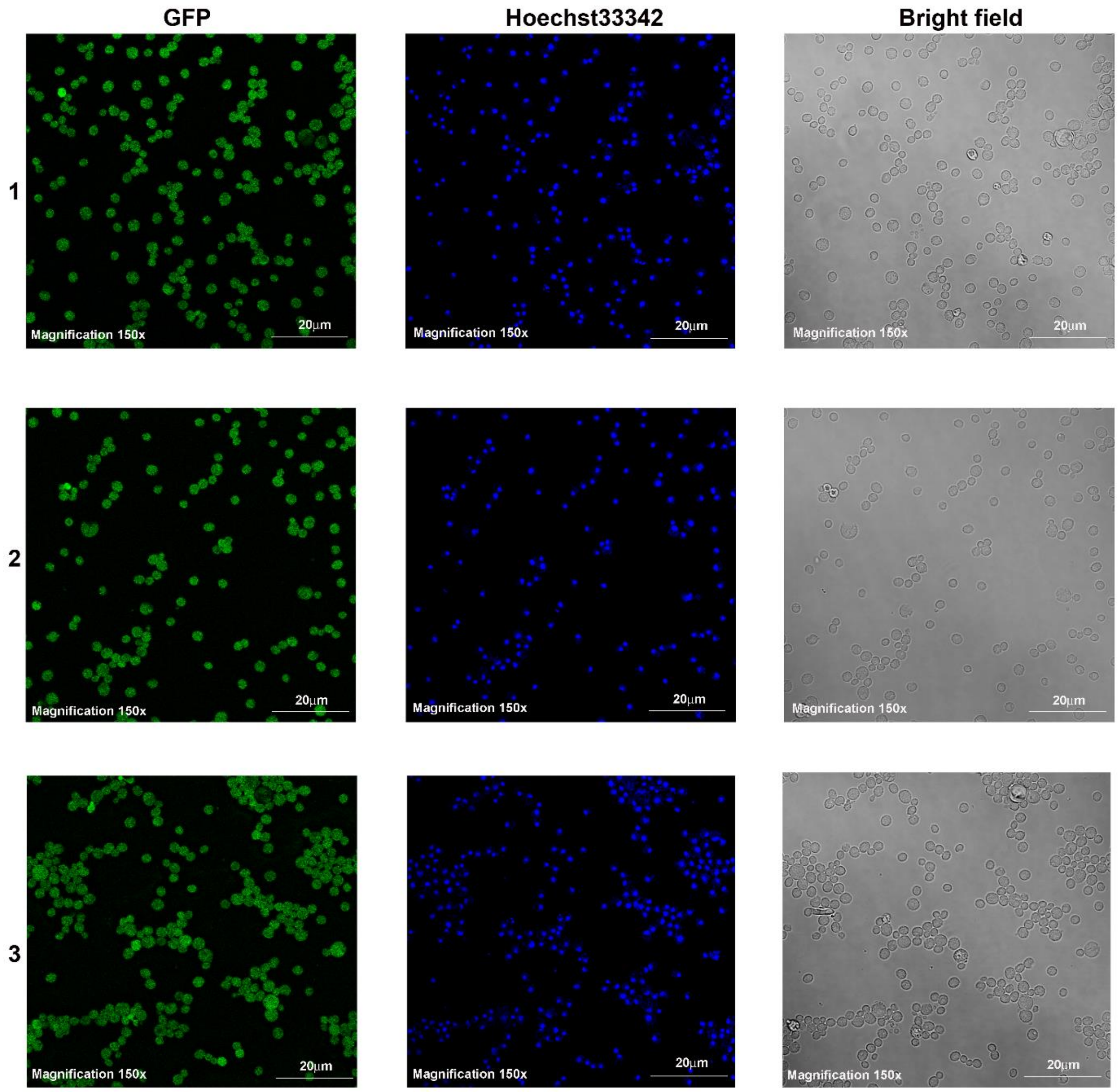

Fig. S3. Subcellular localization of Mxr1N81. Images (GFP, Hoechst 33342, bright field) of three biological replicates (1-3) are shown.

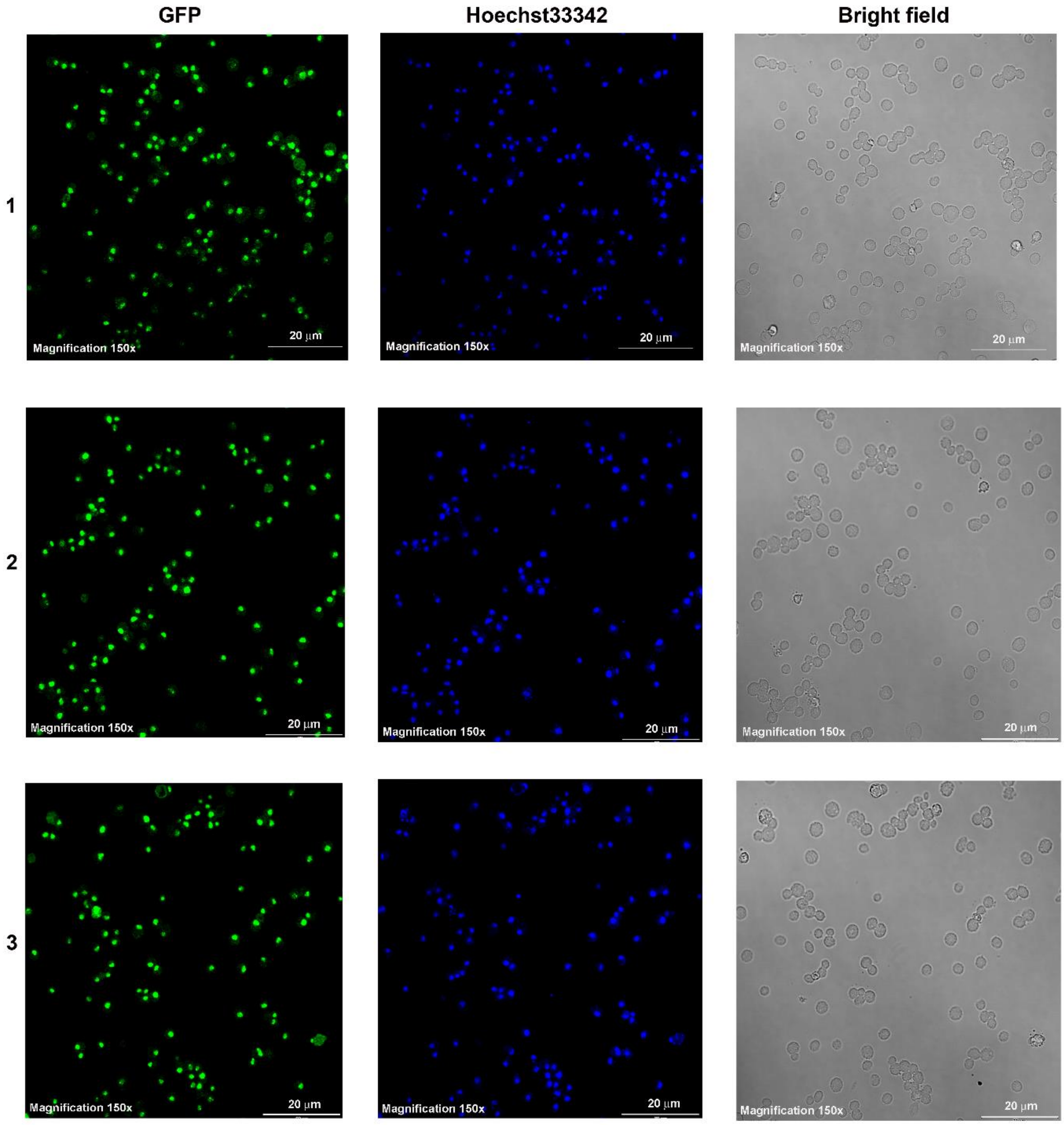

Fig. S4. Subcellular localization of Mxr1N109. Images (GFP, Hoechst 33342, bright field) of three biological replicates (1-3) are shown.

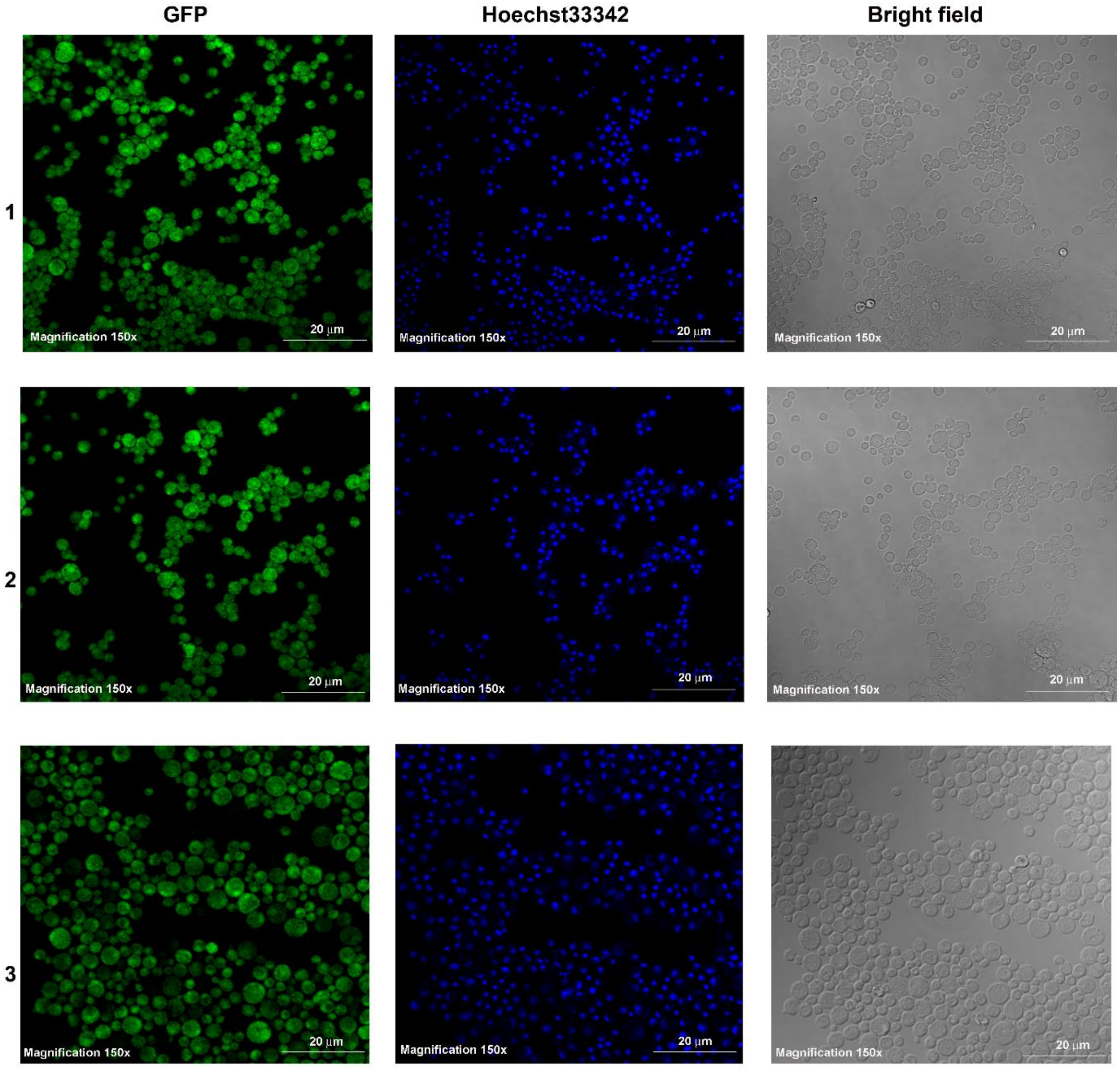

Fig. S5. Subcellular localization of Mxr1N250-M2. Images (GFP, Hoechst 33342, bright field) of three biological replicates (1-3) are shown.

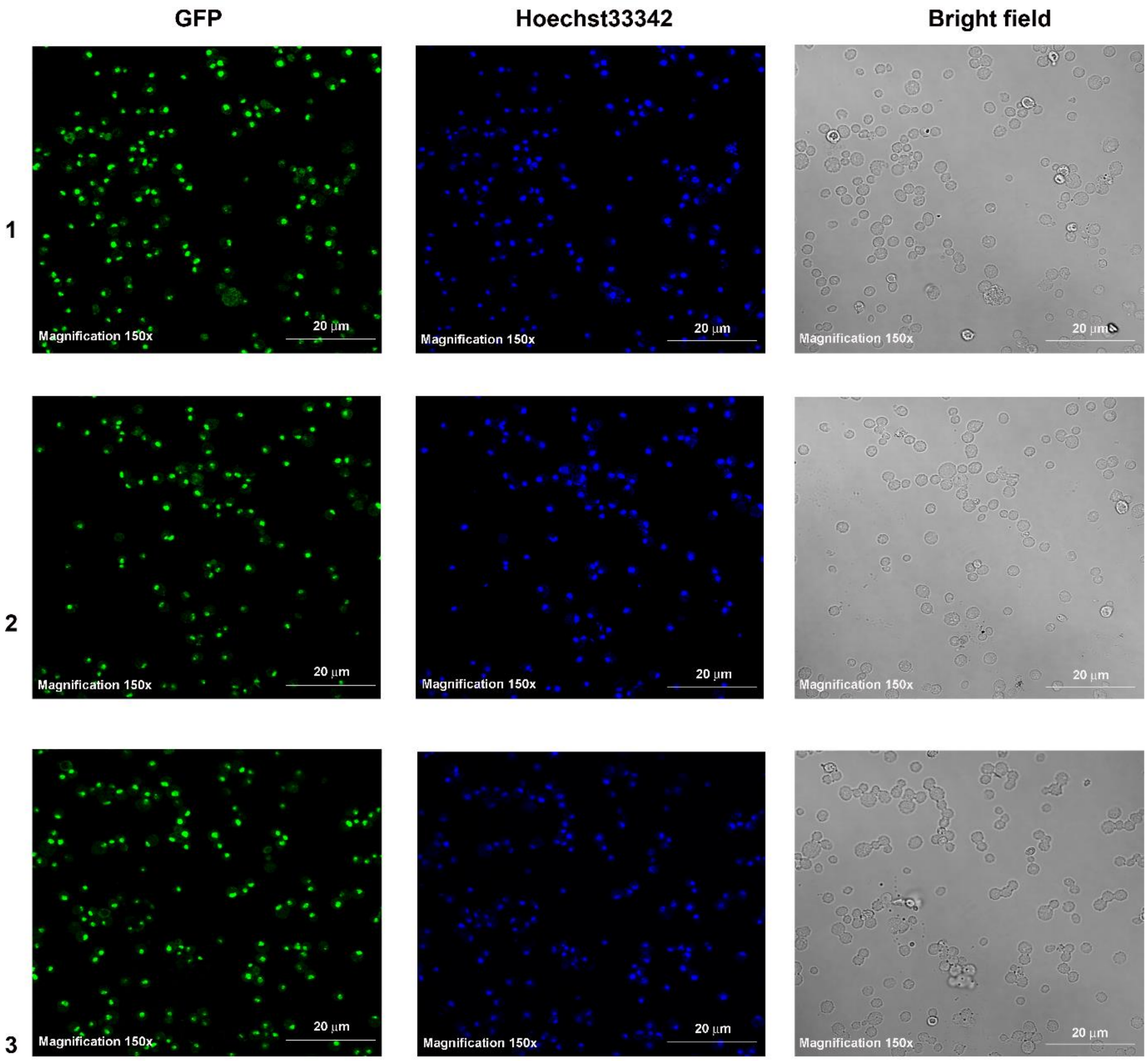

Fig. S6. Subcellular localization of Mxr1N250-M1. Images (GFP, Hoechst 33342, bright field) of three biological replicates (1-3) are shown.

SEQUENCE	START	END	% MATCH
QSVTSEMSQ	431	439	67% match
DSFTNRFLN	493	501	75% match
YSLFKAILD	514	522	92% match
QSLTLSVMY	603	611	92% match
NDISLNVVI	617	625	92% match
NVVIRQVNA	622	630	83% match
NALNSLVKT	629	637	83% match
**TSIIDLFNI**	**643**	**651**	**Perfect match**
NELWNQFVK	657	665	75% match
ELWNQFVKI	658	666	92% match
**DALFTKVVN**	**784**	**792**	**Perfect match**
NVWLKNWNQ	850	858	58% match
**EFVDDMINL**	**883**	**891**	**Perfect match**
**DMINLSLII**	**887**	**895**	**Perfect match**
SLIIIKIMK	892	900	75% match
QELNLTFDN	920	928	67% match
DNFDEKISL	927	935	58% match
TILFDIFLM	938	946	83% match
TDIDEKLDQ	998	1006	67% match
TTLNSVLLA	1073	1081	75% match
**ESFFDFLYE**	**1117**	**1125**	**Perfect match**

Fig. S7. Putative 9 amino acid TADs between 401 and 1155 amino acids of Mxr1p (https://www.med.muni.cz/9aaTAD/index.php)

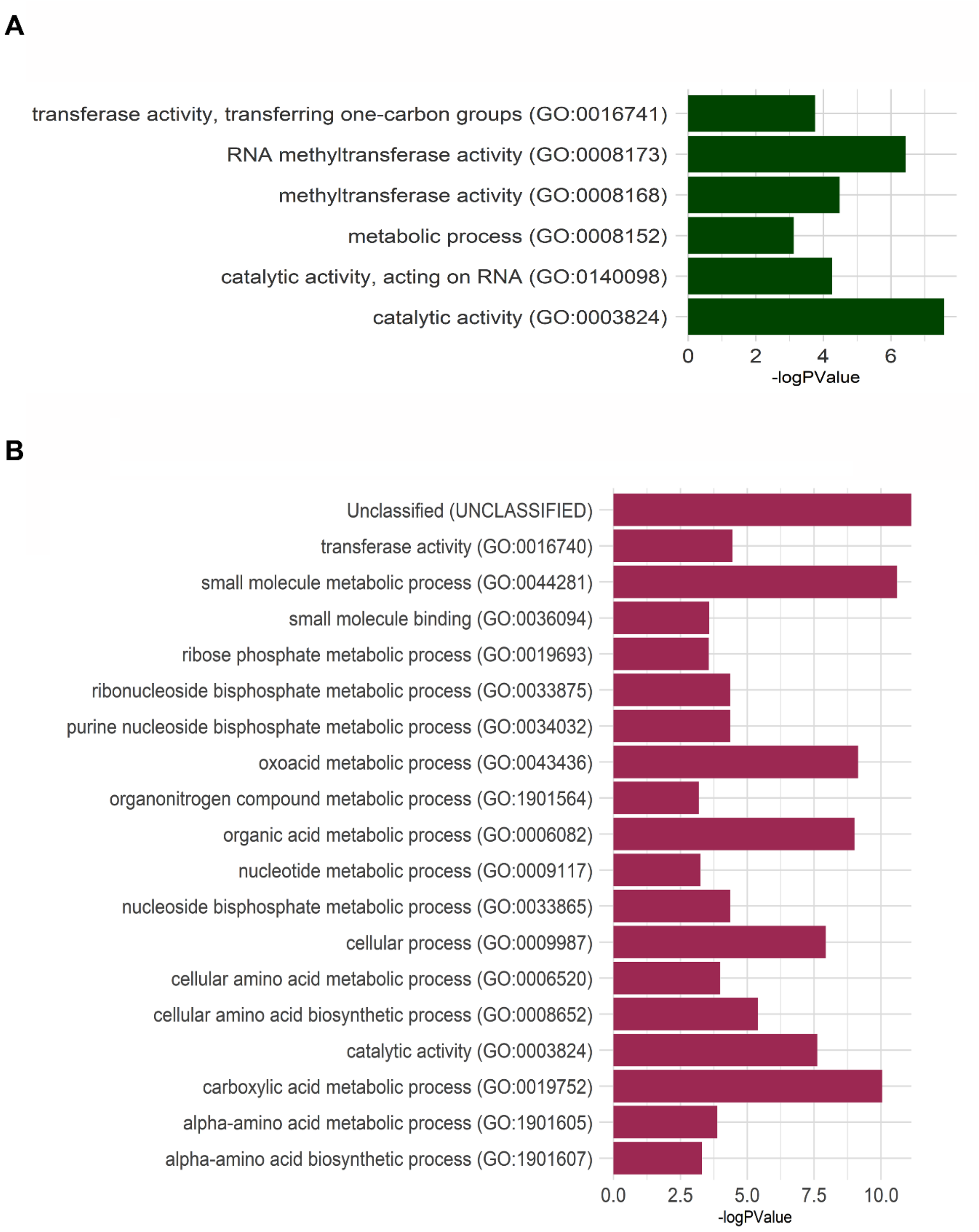

Fig. S8. Enriched gene ontology categories for downregulated (**A**) and upregulated (**B**) genes in *Δmxr1* cultured in YNBM.
